# Biodiversity of Lignicolous Freshwater Hyphomycetes from China and Thailand and Description of Sixteen Species

**DOI:** 10.3390/jof7080669

**Published:** 2021-08-18

**Authors:** Dan-Feng Bao, Kevin D. Hyde, Eric H. C. McKenzie, Rajesh Jeewon, Hong-Yan Su, Sarunya Nalumpang, Zong-Long Luo

**Affiliations:** 1 College of Agriculture and Biological Sciences, Dali University, Dali 671003, China; baodanfeng0922@gmail.com (D.-F.B.); suhongyan16@hotmail.com (H.-Y.S.); 2 Center of Excellence in Fungal Research, Mae Fah Luang University, Chiang Rai 57100, Thailand; kdhyde3@gmail.com; 3 Department of Entomology and Plant Pathology, Faculty of Agriculture, Chiang Mai University, Chiang Mai 50200, Thailand; sarunyav@gmail.com; 4 Research Centre of Microbial Diversity and Sustainable Ultilization, Chiang Mai University, Chiang Mai 50200, Thailand; 5 Innovative Institute of Plant Health, Zhongkai University of Agriculture and Engineering, Haizhu District, Guangzhou 510225, China; 6 Manaaki Whenua Landcare Research, Auckland 92170, New Zealand; mckenziee@landcareresearch.co.nz; 7 Department of Health Sciences, Faculty of Medicine and Health Sciences, University of Mauritius, Reduit 80837, Mauritius; r.jeewon@uom.ac.mu

**Keywords:** *Aquapteridospora bambusinum*, asexual-morph, new species, phylogeny, taxonomy

## Abstract

Freshwater hyphomycetes are a highly diverse group of fungi with a worldwide distribution and have been mostly reported from tropical and subtropical regions. During investigations of freshwater fungi from the Greater Mekong subregion in China and Thailand, sixteen freshwater hyphomycetes (three of them belong to the class Dothideomycetes while thirteen belong to the class Sordariomycetes) were collected. Based on morphology and multi-gene phylogenetic analyses, *Neospadicoides thailandica*, *Pseudodactylaria aquatica*, *Sporidesmium nujiangense*, *Tetraploa thailandica*, *Vamsapriya* *aquatica* and *Wongia fusiformis* are described as new species; *Aquapteridospora bambusinum* is proposed as a new combination; *Acrodictys liputii*, *Chloridium gonytrichii*, *Pseudoberkleasmium chiangmaiense*, *Pleomonodictys capensis*, *Sporidesmium aturbinatum* and *Vamsapriya indica* are reported as new country records; and *Sporidesmium tropicale*, *Sporoschisma chiangraiense* and *Sporoschisma longicatenatum* are introduced as three new collections. In addition, a checklist of freshwater fungi from China over the last five years is also provided.

## 1. Introduction

Freshwater hyphomycetes are anamorphic (asexual) fungi, typically with relatively large branched stauroform or scolecoform conidia [1]. These taxa commonly live on submerged woody debris, decaying tree leaves and roots of riparian vegetation in lotic habitats [1,2,3,4]. Freshwater hyphomycetes are an ecologically defined group that can be divided into four biological groups, namely aero–aquatic hyphomycetes, terrestrial–aquatic hyphomycetes, submerged-aquatic (amphibious) hyphomycetes and Ingoldian fungi [4,5,6,7]. In this study, we focused on submerged-aquatic hyphomycetes, which are defined as fungi growing on submerged decaying wood [6]. Freshwater hyphomycetes are cosmopolitan with most species reported from temperate, tropic and subtropic regions [2]. They play important roles in the ecosystem, decay when submerged and waterlogged, have woody debris and release nutrients, which are imperative in ecosystem functioning [3].

Cecil Terence Ingold was the first mycologist to study freshwater hyphomycetes. He found tetraradiate and sigmoid conidia in foam and reported the connection of these conidia with the submerged leaves [8]. Subsequently, several new genera and new species were recorded from submerged leaves in England [9,10]. Since then, many freshwater fungi have been widely reported, e.g., Australia [11], China [12], Cuba [13], India [14], Japan [15], Malaysia [16,17] and Thailand [18,19]. Until 2013, around 530 freshwater fungi had been reported [20]. Since then, many new species, genera and families of freshwater fungi have been introduced worldwide [21,22,23,24,25,26,27,28,29,30].

Traditional identification of freshwater hyphomycetes was based on morphological characters, especially conidiophores, conidiogenous cells and conidia [6]. However, the morphology of some species is quite similar and some morphological characters overlap between taxa and, therefore, morphology alone is not sufficient for reliable identification at lower taxonomic levels [31,32,33]. Molecular data has significantly solved this problem, allowing a better species classification of fungi [31,32,33,34]. For example, species of the freshwater hyphomycetes genus *Cancellidium*, are quite similar in morphology and previous studies have identified them as the same species. However, Hyde et al. [35] showed that they are not one species, and there are at least four species based on phylogenetic analyses.

Molecular data has significantly improved our understanding on the phylogenetic relationships and taxonomy of freshwater fungi. Phylogenetic analyses showed that freshwater hyphomycetes are polyphyletic and distributed in different phyla, such as Ascomycota and Basidiomycota, the dominant phylum being Ascomycota [6,29,30,36], with most species reported in Dothideomycetes and Sordariomycetes [29,30,37], and a few species belonging to Eurotiomycetes [38,39,40]. Some freshwater hyphomycetes have been linked with their sexual morphs [6,29,41,42,43,44,45].

Freshwater fungi in Thailand have been studied for several decades. Tubaki et al. [46] found 40 Ingoldian fungi from foams in Thailand. Since then, many freshwater species have been described in a series entitled “Lignicolous freshwater Ascomycota from Thailand” [47,48,49,50,51]. Until 2004, 158 freshwater fungal genera had been recorded in Thailand [52]. Zhang et al. [53] provided a checklist of 173 freshwater fungi in Thailand, mainly collected from the north (Chiang Mai and Chiang Rai), south (Narathiwat) and northeast (Nakorn Ratchassima) regions. Many new species and new genera from Thailand have since been described [27,28,29,30,51].

He et al. [54] first reported a freshwater fungus from China when *Vibrissea truncorum* (Alb. & Schwein.) Fr. was found on submerged wood in Guizhou Province. Subsequently, mycologists started to investigate the freshwater fungi from Yunnan Province and Hongkong, and many new taxa have been described [12,29,30,54,55,56,57,58,59,60,61,62,63,64,65,66]; a few have been reported from Guizhou province and Tibet autonomous region [67,68,69]. Freshwater species are continually being introduced from China [29,30,38,45,65,66,70,71,72,73]. Until 2013, 782 freshwater fungal species had been reported from China [30,63], but now, more than 1000 freshwater fungi have been reported from China.

During an investigation of freshwater fungi from the Greater Mekong subregion, sixteen freshwater hyphomycetes were collected from Thailand and southern China (Yunnan Province and Tibet autonomous region), of which six were new species, one new combination, six new geographic records and three new collections are introduced based on both morphology and phylogeny.

## 2. Materials and Methods

### 2.1. Collection, Isolation and Morphology

Samples of submerged decaying wood were collected from streams or rivers in Yunnan and Tibet autonomous region, China and Thailand during 2015–2020. Sample incubation, examination and morphological studies followed the methods provided by Luo et al. [65]. Single spore isolations were made on potato dextrose agar (PDA) and germinating conidia were transferred to fresh PDA [74]. Herbarium materials were deposited in the herbarium of Mae Fah Luang University (MFLU), Chiang Rai, Thailand and herbarium of Cryptogams Kunming Institute of Botany Academia Sinica (Herb. HKAS). Cultures were deposited in Mae Fah Luang University Culture Collection (MFLUCC) and Dali University (DLUCC) China. Faces of Fungi and Index Fungorum numbers were registered as outlined in Jayasiri et al. [75] and Index Fungorum (2021). A check list of freshwater fungi from China has been made based on published data.

### 2.2. DNA Extraction, PCR Amplification and Sequencing

DNA extraction was carried out with an Ezup Column Fungi Genomic DNA Purification Kit (Tsingke Biological Engineering Technology, Kunming, Yunnan, China) based on the manufacturer’s protocol. ITS, LSU, SSU, TEF1-a and RPB2 gene regions were amplified using the primer pairs ITS5/ITS4, LR0R/LR7, NS1/NS4, 983F/2218R and fRPB2-5F/fRPB2-7cR, respectively [76,77,78]. Amplification was performed in 25 μL reaction volume consisting of 1 μL of (10–50 ng) genomic DNA and 1 μL of each primer (10 μM), 9.5 μL ddH_2_O and 12.5 μL 2 × Taq PCR Master Mix with blue dye (Sangon Biotech, China). The amplification condition for ITS, LSU, SSU, TEF1-a and RPB2 were followed [65]. The amplified PCR fragments were sent to a commercial sequencing provider (Tsingke Biological Engineering Technology and Services Co., Kunming, Yunnan, China).

### 2.3. Phylogenetic Analyses

The taxa used in the phylogenetic analyses were obtained from previous studies and downloaded from GenBank. SEQMAN v. 7.0.0 (DNASTAR, Madison, WI, USA). MAFFT v.7 online program (http://mafft.cbrc.jp/alignment/server/, 12 August 2021) was used to assemble the consensus sequences and align the sequences respectively [79]. BioEdit was used to manually adjust the alignments and the alignment fasta file was converted to Phylip format by phylogeny website tool “ALTER” [80].

Maximum likelihood (ML) analysis was performed in RAxML-HPC v.8 [81,82] on the XSEDE Teragrid of the CIPRES Science Gateway (https://www.phylo.org, 12 August 2021) [83] with rapid bootstrap analysis, followed by 1000 bootstrap replicates. The final tree was selected amongst suboptimal trees from each run by comparing likelihood scores under the GTR-Gamma substitution model. Bayesian analyses were performed by MrBayes v. 3.2 [84], best fit model of DNA evolution for the Bayesian inference analysis were estimated by MrModeltest v. 2.2 [85]. Posterior probabilities (PP) [86,87] were defined by the Bayesian Markov Chain Monte Carlo (BMCMC) sampling method in MrBayes v. 3.0b4 [88]. Phylogenetic trees were visualized with FigTree v. 1.4.2 [89] and modified in Adobe Illustrator CS5 software (Adobe Systems, San Jose, CA, USA). Newly obtained sequences in this study were deposited in GenBank.

## 3. Results

### 3.1. Taxonomy

#### 3.1.1. Dothideomycetes O.E. Erikss. and Winka, Myconet 1(1): 5 (1997)

**Figure 1:**
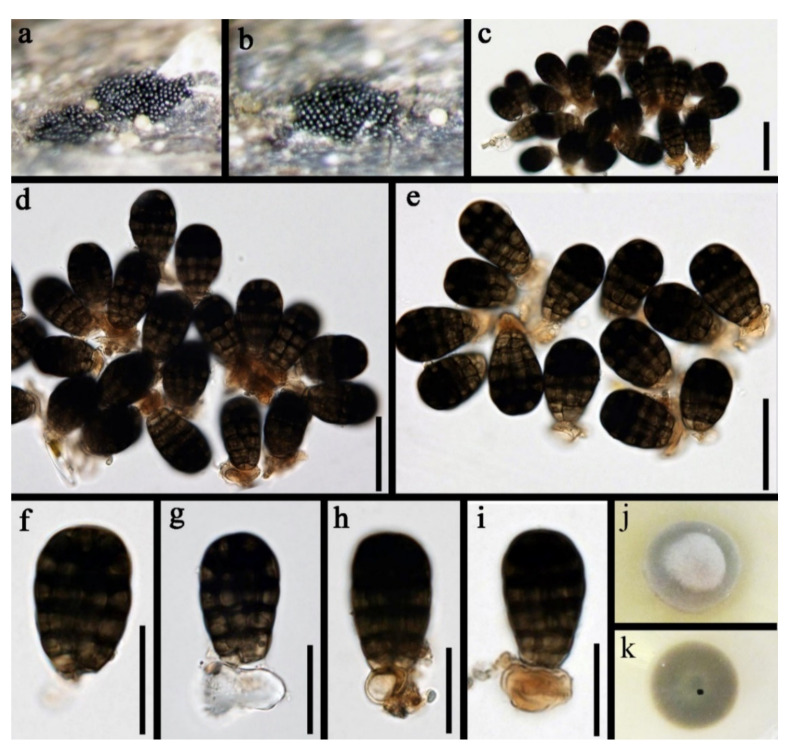
*Pseudoberkleasmium chiangmaiense* (HKAS 115794). (**a**,**b**) Colony on submerged decaying wood; (**c**–**i**) Conidia with basal cell; (**j**,**k**) Colonies on PDA from above and below. Scale bars: (**c**–**e**) 30 µm; (**f**–**i**) 20 µm.

Pleosporales Luttr. ex M.E. Barr 1987
*Pseudoberkleasmiaceae* Phukhams. and K.D. Hyde
*Pseudoberkleasmium* Tibpromma and K.D. Hyde
*Pseudoberkleasmium chiangmaiense* Y.Z. Lu and K.D. Hyde, in Hyde et al., Fungal Diversity 96:38 (Figure 1).

*Saprobic* on submerged decayed wood in freshwater habitats. **Sexual morph**: Undetermined. **Asexual morph**: *Colonies* on substratum superficial, sporodochia, scattered, compact, irregular, black and shining. *Mycelium* immersed and composed of hyaline to pale brown, branched, septate and smooth-walled hyphae. *Conidiophores* mononematous and micronematous, reduced to conidiogenous cells, hyaline to pale brown and smooth-walled. *Conidiogenous cells* monoblastic, holoblastic, determinate, terminal, globose to subglobose, hyaline when young and brown to dark brown when mature. *Conidia* 17–30 × 19–35 µm, (x = 33 × 18 μm, *n* = 30) solitary, acrogenous, muriform, obovoid to broadly ellipsoidal, flattened, dark brown to black, paler towards the base and smooth with a hyaline to brown basal cell.

Culture characteristics: Conidia germinating on PDA within 24 h. Colonies grew on PDA, reaching 20–30 mm in three weeks at 25 °C. Mycelia superficial, circular, with the entire margin flat and smooth from above, white at the center, pale grey at the edge, greyish brown from below and not producing pigmentation in culture.

Material examined: Lancang River, Yunnan Province, China, saprobic on submerged decaying wood, 12 November 2017, Z.L. Luo, S-1655 (HKAS 115794), living culture, DLUCC 1655. GenBank accession numbers: (LSU) MZ420759, (SSU) MZ420749, (ITS) MZ420744, (tef-1) MZ442693.

**Figure 2:**
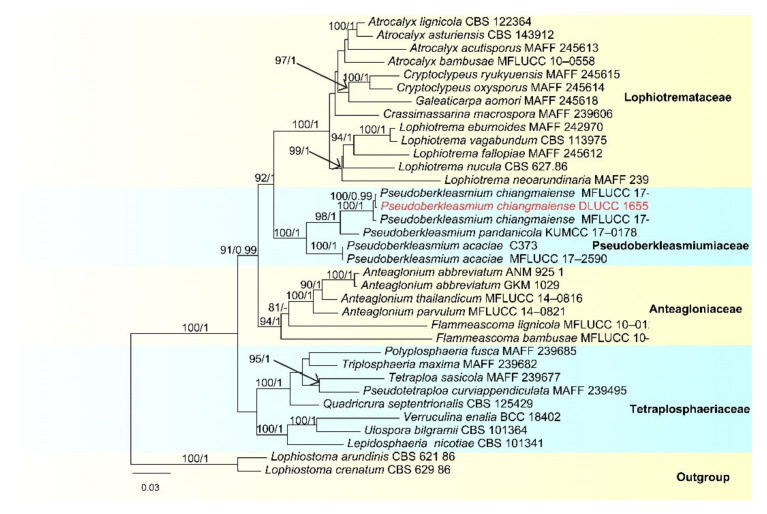
RAxML tree based on analysis of combined LSU, SSU, ITS, TEF1-a and RPB2 dataset. The combined dataset comprises 65 strains with 4833 characters including gaps (LSU: 1300 bp, SSU: 1036 bp, ITS: 528 bp, TEF1-a: 957 bp, RPB2: 1012 bp). The tree is rooted with *Lophiostoma crenatum* (CBS 629.86) and *L. arundinis* (CBS 621.86). Tree topology of the maximum likelihood analysis and Bayesian analysis are similar. The RAxML analysis of the combined dataset yielded a best scoring tree (Figure 2) with a final ML likelihood value of −24484.351178. The matrix had 1464 distinct alignment patterns, with 26.42% undetermined characters or gaps. Estimated base frequencies were as follows: A = 0.248305, C = 0.251085, G = 0.269428, T = 0.231183; substitution rates AC = 1.513073, AG = 3.989931, AT = 1.420330, CG = 1.195982, CT = 9.915363, GT = 1.000000; gamma distribution shape parameter α = 0.160321. Bootstrap values for ML ≥ 75% and Bayesian posterior probabilities (PP) ≥ 0.95 are labelled on the nodes. The ex-type strains are in bold and black. The newly obtained sequence is indicated in red.

Notes: *Pseudoberkleasmium chiangmaiense* was introduced by Hyde et al. [90] from Thailand. Phylogenetic analyses showed that our new isolate (DLUCC 1655) clustered with the ex-type strain of *P. chiangmaiense* (MFLUCC 17–1809) with strong bootstrap support (100 ML/1.00 PP, Figure 2). Morphology of our new isolate is almost identical to the holotype of *P. chiangmaiense*, except the basal cell of conidia in our isolate are hyaline to brown, while in the holotype they are hyaline. Based on both phylogeny and morphology, we identified our isolate as *P. chiangmaiense*, which is new to China.

**Figure 3:**
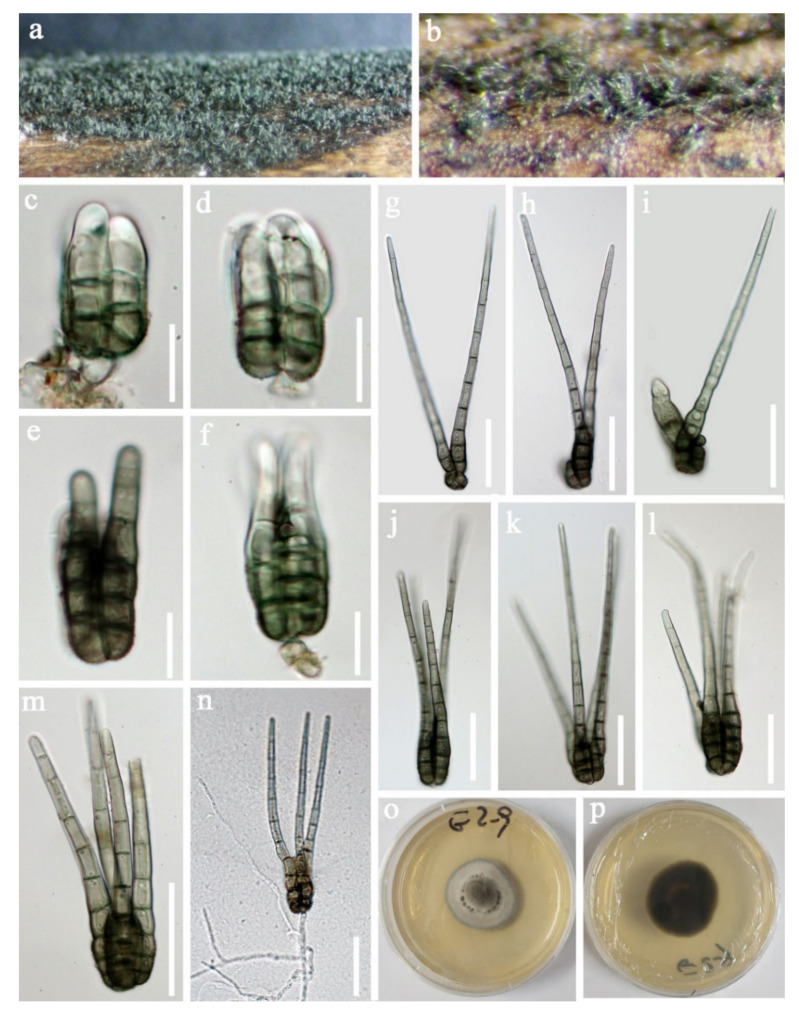
*Tetraploa thailandica* (MFLU 21–0030, holotype). (**a**,**b**) Colonies on submerged wood; (**c**–**m**) Conidia; (**n**) Germinating conidium; (**o**,**p**) Colonies on MEA from above and below. Scale bars: (**c**–**f**) 15 µm; (**g**–**n**) 40 µm.

Tetraplosphaeriaceae Kaz. Tanaka and K. Hiray
*Tetraploa* Berk. and Broome
*Tetraploa thailandica* D.F. Bao, H.Y. Su, K.D. Hyde and Z.L. Luo, sp. nov. (Figure 3).
Index Fungorum number: IF558591; *Facesoffungi number*: FoF 09915
Holotype: MFLU 21–0030
Etymology—Referring to Thailand, where the fungus was collected.

*Saprobic* on submerged decaying wood. **Sexual morph:** Undetermined. **Asexual morph:** *Colonies* effuse and brown or dark greyish brown. *Mycelium* mostly immersed, composed of branched, septate, subhyaline and hyphae. *Conidiophores* indistinct. *Conidiogenous cells* holoblastic, monoblastic or occasionally polyblastic, integrated, determinate, terminal or intercalary and cylindrical. *Conidia* solitary, dry, straight, septate, verrucose and composed of a conidial body at the base with 2–4 brown to pale brown apical appendages. *Conidial body* 23–37 × 17–22.5 µm (x = 30 × 20 μm, *n* = 30) narrowly ovate or ovate, greyish-brown, pale brown to subhyaline at the apex when young, and brown to dark brown at maturity, verrucose and composed of 2–4 closely-adhered vertical columns of cell, with each column 3–5-septate. *Appendages* 73–136 µm long, 2–3.5 µm wide at the apex and 5.5–8 µm at the base (*n* = 35). Setose brown to greyish brown, 5–10-pseudoseptate and smooth-walled.

Culture characteristics: Conidia germinating on PDA within 24 h. Germ tubes were produced from the basal cell of conidia. Colonies grew on MEA, reaching 25–35 mm in three weeks at 25 °C. Mycelia was superficial and circular with the entire margin flat and smooth, greyish brown from above, dark brown to black from below and not producing pigmentation in culture.

Material examined: Sakon Nakhon, Tao Ngoi, Thailand., saprobic on submerged decaying wood, 12 November 2017, D.F. Bao, B144 (MFLU 21–0030, holotype), ex-type living culture, MFLUCC 21–0030. GenBank accession numbers: (LSU) MZ412530, (SSU) MZ413274, (ITS) MZ412518.

Notes: species of *Tetraploa* are quite similar and the asexual morphs are characterized by micronematous conidiophores, monoblastic conidiogenous cells and tetraploate conidia composed of four columns, which are short-cylindrical, euseptate, brown, verrucose at the base and with 2–4-setose and septate appendages at the apex [91,92,93]. *Tetraploa thailandica* is morphologically most similar to *T*. *aquatica*. They share some characteristicss, such as monoblastic or occasionally polyblastic conidiogenous cells and conidia composed of four closely-adhered vertical columns of cells, with 2–4 apical appendages. However, *T. aquatica* has a shorter but wider conidial body (23–37 × 17–22.5 vs. 22.5–27 × 20–24). Moreover, the vertical columns of *T. thailandica* are 3–5-septate, while those of *T. aquatica* are 2–3-septate [92].

**Figure 4:**
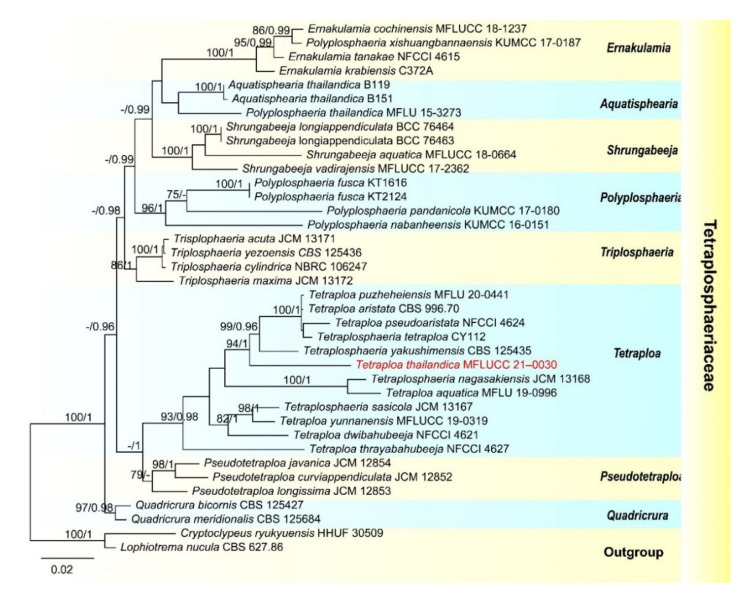
RAxML tree based on analysis of combined ITS, LSU and SSU dataset. The combined analyses include 38 strains with 2673 characters including gaps (LSU: 1064 bp, SSU: 1013 bp and ITS: 596). The tree is rooted with *Cryptoclypeus ryukyuensis* (HHUF 30509) and *Lophiotrema nucula* (CBS 627.86). Tree topology of the maximum likelihood analysis and Bayesian analysis are similar. The RAxML analysis of the combined dataset yielded a best scoring tree (Figure 4) with a final ML likelihood value of −10303.690850. The matrix had 614 distinct alignment patterns, with 21.18% undetermined characters or gaps. Estimated base frequencies were as follows: A = 0.249102, C = 0.235801, G = 0.278062, T = 0.237035; substitution rates AC = 3.429408, AG = 3.446748, AT = 1.814192, CG = 1.255022, CT = 8.630115, GT = 1.000000; gamma distribution shape parameter α = 0.117960. Bootstrap values for ML ≥ 75% and Bayesian posterior probabilities (PP) ≥ 0.95 are labelled on the nodes. The newly obtained sequence is indicated in red.

In our phylogenetic analyses, *Tetraploa thailandica* formed a distinct lineage within the genus and was close to *T. yakushimensis* (Figure 4). However, *T. thailandica* differs from *T. yakushimensis* in having 3–5-celled and narrower conidia (17–22.5 vs. 20–30 µm) composed of 2–4 closely-adhered vertical columns of cell and 2–4 setose appendages. Conidia of *T. yakushimensis* are 4-celled, composed of four columns and four appendages [91]. Moreover, *T. thailandica* has more septa in the appendages than *T. yakushimensis* (5–10 vs. 3–8).

**Figure 5:**
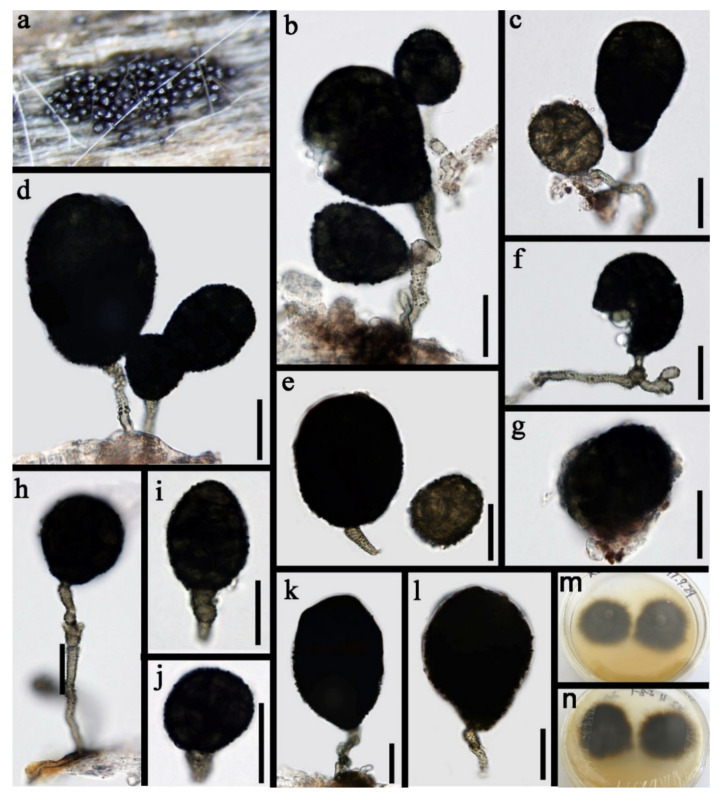
*Pleomonodictys capensis* (HKAS 115793). (**a**) Colony on submerged decaying wood; (**b**–**f**,**h**–**l**) Conidiophores with attached conidia; (**g**) Conidia. (**m**,**n**) Colonies on MEA from above and below. Scale bars: (**b**–**l**) 20 µm.

Pleomonodictydaceae Hern.-Restr., J. Mena and Gené
*Pleomonodictys* Hern.-Restr., J. Mena and Gené
*Pleomonodictys capensis* (R.C. Sinclair, Boshoff and Eicker) Hern.-Restr., J. Mena and Gené, Studies in Mycology 86:77 (2017) (Figure 5).

*Saprobic* on submerged decayed wood in freshwater habitats. **Sexual morph:** Undetermined. **Asexual morph**: *Colonies* effuse on natural substrate, scattered, punctiform, dark brown to black and glistening. *Mycelium* mostly immersed and composed of branched verrucose, septate, brown to pale brown and hyphae. *Conidiophores* 4–6.5 μm wide (x = 5.0 μm, *n* = 20), mononematous, micronematous to semi-macronematous, cylindrical, straight or flexuous, septate, branched or unbranched, pale brown to brown and verrucose. *Conidiogenous cells* polyblastic, discrete, intercalary and brown. *Conidia* 28.5–60 × 23.5–44 μm (x = 44 × 33.7 μm, *n* = 30), pleurogenous, solitary, ellipsoid to subglobose, obovoid, pyriform to broadly clavate, muriform, dark brown to black with base rounded or truncate and thick-walled.

Culture characteristics: Conidia germinating on PDA within 24 h. Colonies grew on MEA, reaching 25–30 mm in four weeks at 25 °C, with rough surface, dense mycelia, velvety, dry, dark brown from above, dark brown to black from below; not producing pigmentation in culture.

Material examined: China, Tibet autonomous region, on submerged decaying wood, May 2017, Z.L. Luo, S-1323 (HKAS 115793), living culture, DLUCC 1323. GenBank accession numbers: (LSU) MZ420757, (ITS) MZ420742, (RPB2) MZ442696.

Notes: *Pleomonodictys* was established for two *monodictys*-like species, *Pleomonodictys capensis* and *P. descalsii* [94]. The genus is characterized by micronematous or semi-macronematous conidiophores, often reduced to conidiogenous loci on the hyphae and blastic conidia which are solitary or in short chains, variable in shape, muriform, dark brown to black. Our species fits well within the species concept of *Pleomonodictys*. 

Morphologically, the new collection (MFLUCC 18–1499) is almost identical to the holotype of *P. capensis*, such as micronematous to semi-macronematous, pale brown to brown, verrucose conidiophores and muriform, obovoid, ellipsoid to subglobose, broadly clavate to pyriform, dark brown to black conidia and conidial size of our collection overlapping with the holotype (28.5–60 × 23.5–44 vs. 30–100 × 17–60 μm) [95]. Thus, we identified our collection (MFLUCC 18–1499) as *P. capensis*. *Pleomonodictys capensis* was introduced by Sinclair et al. [95], collected from terrestrial habitats in South Africa. Our collection was collected from freshwater habitat in China, a new record for China.

**Figure 6:**
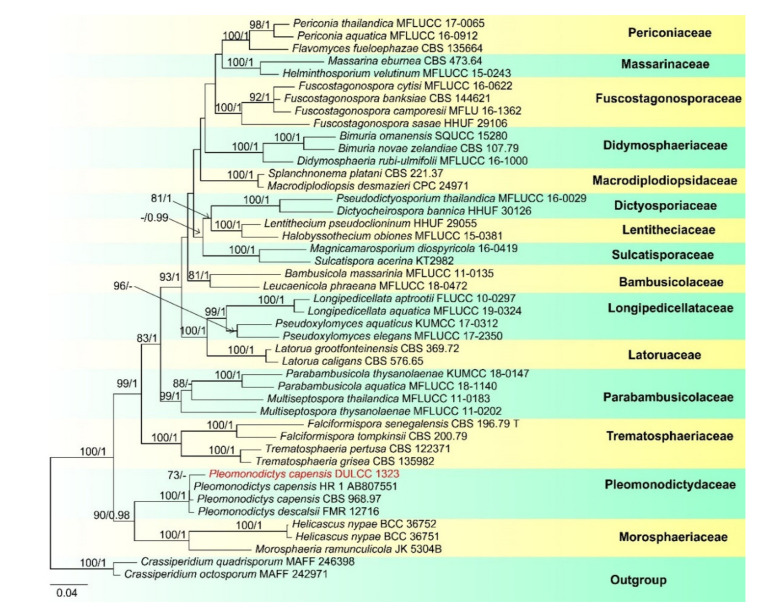
RAxML tree based on analysis of combined LSU, ITS, SSU, TEF and RPB2 dataset. T The combined analyses include 48 strains with 4622 characters including gaps (LSU: 933 bp, ITS: 633 bp SSU: 1040 bp, TEF: 930 bp and RPB2: 1086 bp). The tree is rooted with *Crassiperidium quadrisporum* (MAFF 246398) and *C. octosporum* (MAFF 242971). Tree topology of the maximum likelihood analysis and Bayesian analysis are similar. he RAxML analysis of the combined dataset yielded a best scoring tree (Figure 6) with a final ML likelihood value of −31661.985748. The matrix had 1955 distinct alignment patterns, with 41.80% undetermined characters or gaps. Estimated base frequencies were as follows: A = 0.244236, C = 0.244678, G = 0.272346, T = 0.238739; substitution rates AC = 1.552502, AG = 3.228919, AT = 1.382042, CG = 1.340908, CT = 6.470873, GT = 1.000000; gamma distribution shape parameter α = 0.204896. Bootstrap values for ML ≥ 75% and Bayesian posterior probabilities (PP) ≥ 0.95 are labelled on the nodes. The newly obtained sequence is indicated in red.

Our phylogenetic analyses showed that the strains of *P. capensis* and *P. descalsii* clustered together with high bootstrap support (100 ML/1.00 PP, Figure 6). Hernandez-Restrepo et al. [94] introduced *P. descalsii* almost based on the conidial size, which is much smaller than *P. capensis*. However, the conidial size of our collection is quite similar to *P. descalsii* (28.5–60 × 23.5–44 vs. 28–70 × 24–54 μm) and other morphological characters are indistinguishable. Thus, future morphology and molecular studies are required to confirm the relationship between these two species.

#### 3.1.2. Sordariomycetes

**Figure 7:**
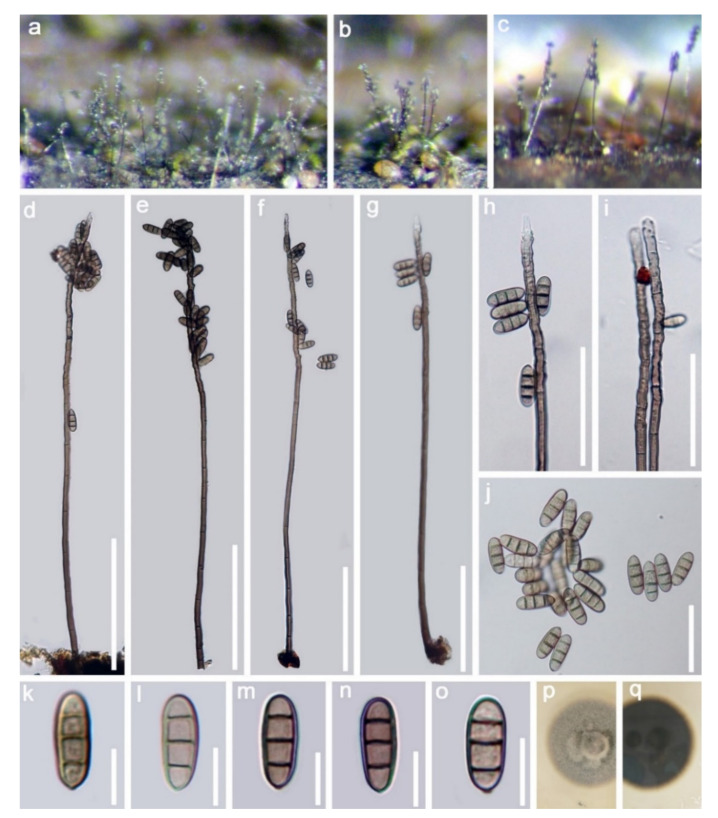
*Aquapteridospora bambusinum* (MFLU 21–0027). (**a**–**c**) Colonies on submerged decaying wood; (**d**–**g**) Conidiophores with conidia; (**h**,**i**) Conidiogenous cells with attached conidia; (**j**–**o**) Conidia; (**p**,**q**) Culture on PDA from above and below. Scale bars: (**d**–**g**) 100 µm; (**h**,**i**) 50 µm; (**j**) 30 µm; (**k**–**o**) 10 µm.

Diaporthomycetidae
Distoseptisporales Z.L. Luo, K.D. Hyde and H.Y. Su
Aquapteridosporaceae K.D. Hyde and Hongsanan
*Aquapteridospora* J. Yang, K.D. Hyde and Maharachch
*Aquapteridospora bambusinum* (D.Q. Dai and K.D. Hyde) D.F. Bao, com. nov. (Figure 7).
*≡Pleurophragmium bambusinum* D.Q. Dai and K.D. Hyde, in Dai et al., Fungal Diversity 82:92
Index Fungorum number: IF 558592

*Saprobic* on submerged decayed wood in freshwater habitats. **Sexual morph:** Undetermined. **Asexual morph:** *Colonies* on substratum, effuse, hairy and brown to dark brown. *Mycelium* partly immersed and composed of septate, branched, smooth and dark brown hyphae. *Conidiophores* 615–715 × 9–13 μm (x = 695.5 × 11 μm, *n* = 20), macronematous, mononematous, erect, simple, septate, straight or slightly flexuous, dark brown, paler to subhyaline towards apex and smooth. *Conidiogenous cells* polyblastic, sympodial, denticulate, integrated, terminal and hyaline to pale brown. *Conidia* 15–18 × 5.5–7 μm (x = 16.5 × 6.5 μm, *n* = 35) acrogenous, solitary, ellipsoid to fusiform with round ends, straight, slightly narrow towards the base, pale brown to dark brown, 3-septate, slightly constricted at the septa, thick-walled and smooth.

Culture characteristics: Conidia germinating on PDA within 24 h. Colonies grew on PDA, reaching 20–25 mm in two weeks at 25 °C. Colonies were medium sparse, circular, flat, a slightly rough surface with edge, entire margin well-defined, grey to brown from above, dark brown to black from below and not producing pigmentation in culture. 

Material examined: Amphoe Thai Charoen Province, Thailand, on submerged decaying wood, acquired on 13 November 2018, D.F. Bao, B113 (MFLU 21–0027), living culture, MFLUCC 21–0027. GenBank accession numbers: (LSU) MZ412526, (SSU) MZ413270, (ITS) MZ412514, (tef-1) MZ442688.

**Figure 8:**
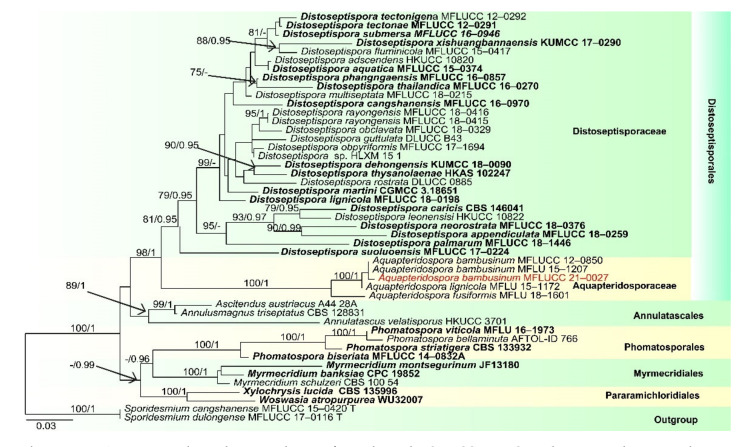
RAxML tree based on analysis of combined LSU, SSU, ITS and TEF1-a dataset. The combined analyses include 47 strains with 3464 characters including gaps (LSU: 943 bp, SSU: 1036 bp, ITS: 528 bp and TEF1-a: 957 bp). The tree is rooted to *Sporidesmium cangshanense* (MFLUCC 15–0420) and *S. dulongense* (MFLUCC 17–0116). Tree topology of the maximum likelihood analysis Bayesian analysis are similar. The RAxML analysis of the combined dataset yielded a best scoring tree (Figure 8) with a final ML likelihood value of −17099.345044. The matrix had 1090 distinct alignment patterns, with 48.94% undetermined characters or gaps. Estimated base frequencies were as follows: A = 0.241037, C = 0.253983, G = 0.288692, T = 0.216288; substitution rates AC = 1.213787, AG = 2.314464, AT = 1.495483, CG = 1.069500, CT = 6.672100, GT = 1.000000; gamma distribution shape parameter α = 0.250596. Bootstrap values for ML ≥ 75% and Bayesian posterior probabilities (PP) ≥ 0.95 are labelled on the nodes. The ex-type strains are in bold and black. The newly obtained sequence is indicated in red.

Notes: *Pleurophragmium bambusinum* was described by Dai et al. [96] and was placed in Sordariomycetes *incertae sedis*. Their phylogenetic analyses showed that *P. bambusinum* clustered with *Ellisembia adscendens* in Sordariomycetes, genera *incertae sedis*. However, in our analyses, *P. bambusinum* was placed in Aquapteridosporaceae and clustered with *Aquapteridospora* species with strong support (100 ML/1.00 PP, Figure 8). *P. bambusinum* is characterized by mononematous, macronematous, simple conidiophores, polyblastic, denticulate, sympodial conidiogenous cells and ellipsoid, brown, 3-septate, conidia. *Pleurophragmium bambusinum* shares some similar characters with *Aquapteridospora*, such as integrated, polyblastic, terminal conidiogenous cells and acrogenous, fusiform, 3-septate conidia. Based on both phylogeny and morphology, we transferred *P. bambusinum* to *Aquapteridospora* and synonymized *Aquapteridospora bambusinum* instead of *P. bambusinum*. 

In our phylogenetic analyses, our new isolate clustered with two strains of *Aquapteridospora bambusinum* with high bootstrap support (Figure 8). Morphology of our collection is identical to *A. bambusinum*. Therefore, we identified our new collection as *A. bambusinum* and it is phylogenetically close to *A. lignicola* but differs by longer conidiophores (615–715 × 9–13 vs. 70–200 × 4–7 μm) and pale brown to dark brown, ellipsoid to fusiform conidia with round ends, lacking a sheath. Conidia of *A. lignicola* are fusiform with obtuse ends, central cells are pale to dark brown and end cells are subhyaline, with a conspicuous sheath [27].

**Figure 9:**
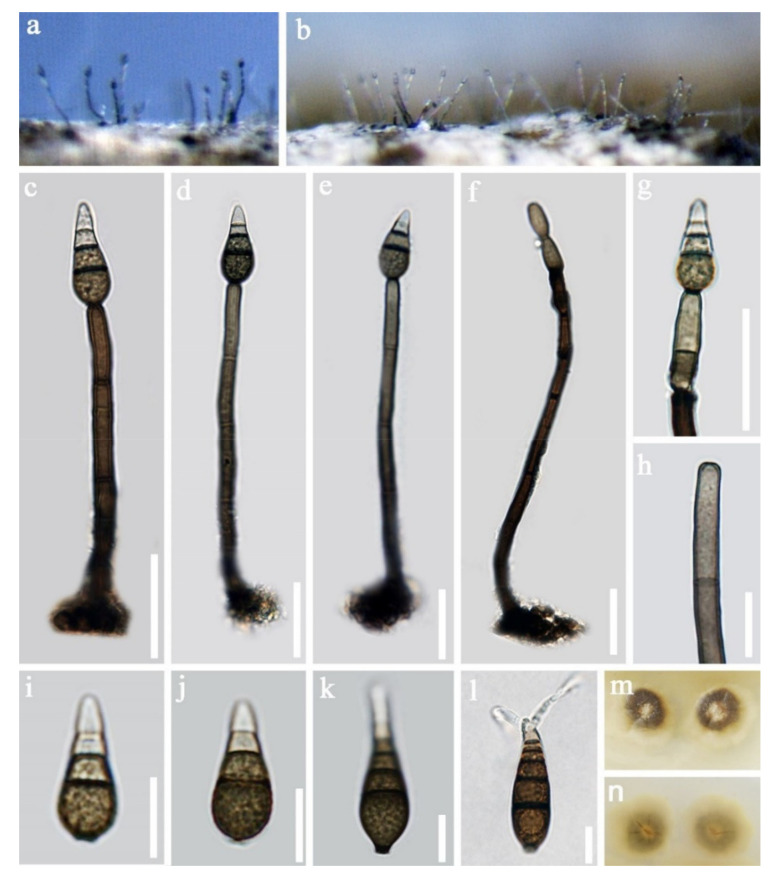
*Sporidesmium aturbinatum* (HKAS 115800). (**a**,**b**) Colonies on submerged decaying wood; (**c**–**e**) Conidiophores with conidia; (**f**) Conidiophores; (**g**) Conidiogenous cells with attached conidium; (**h**) Conidiogenous cells; (**i**–**k**) conidia; (**l**) Germinating conidia. (**m**,**n**) Culture on PDA from above and below. Scale bars: (**c**–**g**) 20 µm; (**h**–**l**) 10 µm.

Sporidesmiales Crous
Sporidesmiaceae Fr., Summa veg
*Sporidesmium* Link, Mag. Gesell. naturf.
*Sporidesmium aturbinatum* (S. Hughes) M.B. Ellis, Mycol. Pap. 70: 49 (1958). (Figure 9).

*Saprobic* on submerged decaying wood. **Sexual morph**: Undetermined. **Asexual morph:** *Colonies* effuse, hairy, dark brown to black and glistening. *Mycelium* partly immersed in the substratum, partly superficial, composed of septate, branched and brown with smooth hyphae. *Conidiophores* 65–111 × 4–5 µm (x = 88.5 × 4.5 μm, *n* = 25), macronematous, mononematous, solitary or sometimes in a small group, cylindrical, straight or slightly flexuous, with up to three successive profilerations, septate, dark brown and paler towards the apex. *Conidiogenous cells* 11–28 × 4–5 µm (x = 14.5 × 4.5 μm, *n* = 25), integrated, terminal, holoblastic, monoblastic, cylindrical and sometimes with percurrent proliferation through the scar. *Conidia* 16–28 × 7–10 µm (x = 21.5 × 8.5 μm, *n* = 25), acrogenous, solitary, dry, pyriform, rostrate, tapering to the apex, 1.5–2.5 µm wide at apex, truncate and 2.8–3.6 µm wide at base, straight or slightly curved, 3(–4)-septate, slightly constricted at septa, two upper cells subhyaline to hyaline, lower cells dark brown and verrucose.

Culture characteristics Conidia germinating on PDA within 24 h. Colonies growing on PDA, reaching 15–20 mm in two weeks at 25 °C, surface rough, with dense mycelia, dry, rigid, from above greyish white at the center, brown at the edge, from below yellowish brown at the center, greyish brown at the edge and not producing pigmentation in culture.

Material examined: Gaoligongshan Mountain, Yunnan Province, China, on submerged decaying wood, in August 2016, H.W. Shen, S1417 (HKAS 115800), living culture, DLUCC 1417. GenBank accession numbers: (LSU) MZ420758, (ITS) MZ420743, (tef-1) MZ442692, (RPB2) MZ442697.

**Figure 10:**
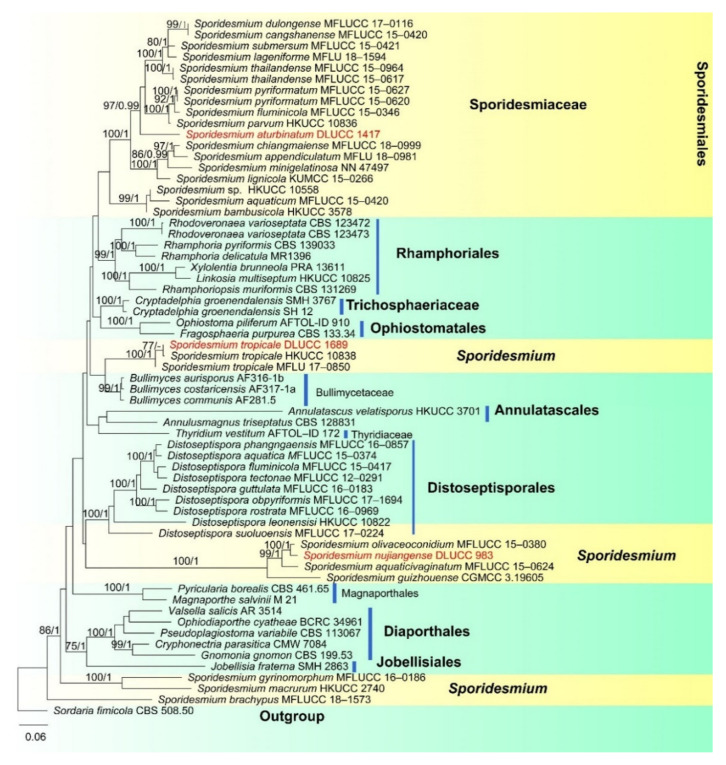
RAxML tree based on analysis of combined LSU, SSU, ITS TEF1-a and RPB2 dataset. The combined analyses include 62 strains with 4832 characters including gaps (LSU: 967 bp, SSU: 1235 bp, ITS: 624 bp TEF1-a: 935 bp and RPB2: 1071 bp). The tree is rooted to *Sordaria fimicola* (CBS 508 50). Tree topology of the maximum likelihood analysis and Bayesian analysis are similar. The RAxML analysis of the combined dataset yielded a best scoring tree with a final ML likelihood value of −44,590.479128. The matrix had 2439 distinct alignment patterns, with 50.13% undetermined characters or gaps. Estimated base frequencies were as follows: A = 0.244673, C = 0.255940, G = 0.282757, T = 0.216629; substitution rates AC = 1.228225, AG = 2.563347, AT = 1.222728, CG = 1.236937, CT = 5.801299, GT = 1.000000; gamma distribution shape parameter α = 0.277316. Bootstrap values for ML ≥ 75% and Bayesian posterior probabilities (PP) ≥ 0.95 are labelled on the nodes. The newly obtained sequences are indicated in red.

Notes: *Sporidesmium aturbinatum* was introduced by Ellis [97] and is characterized by conidiophores that are mononematous, macronematous, solitary or in groups, with up to three successive profilerations and septate, conidiogenous cells that are integrated, terminal, monoblastic and conidia that are pyriform, 2–4-septate, upper cells being subhyaline to hyaline and smooth-walled, lower cells being dark brown andverrucose. Our new collection fits well with the original description of *S. aturbinatum* [97]. Phylogenetic analysis showed that *S. aturbinatum* formed a distinct lineage within Sporidesmiaceae (Figure 10). *Sporidesmium aturbinatum* has been collected from England and Ireland on rotten wood of *Sambucus* and dead culms of *Ammophila*, respectively [97,98]. Our collection was from freshwater habitats in China.

**Figure 11:**
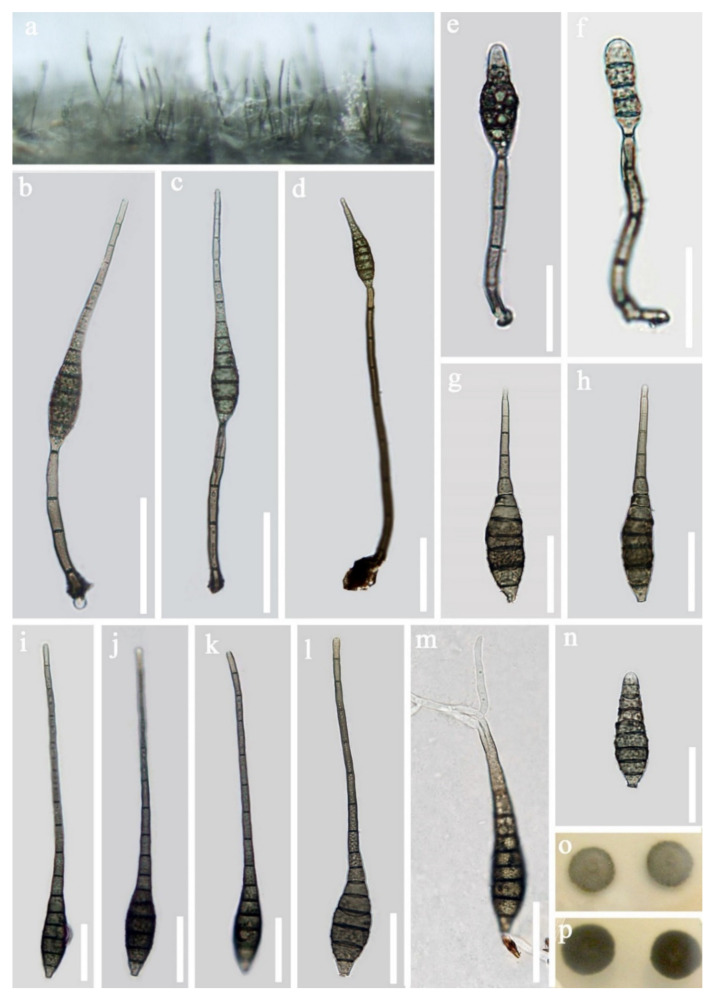
*Sporidesmium tropicale* (HKAS 115799). (**a**) Colonies on submerged decaying wood; (**b**–**f**) Conidiophores with conidia; (**g**–**l**,**n**) Conidia; (**m**) Germinating conidium; (**o**,**p**) Cultures on MEA from above and below. Scale bars: (**b**–**d**) 50 µm; (**e**–**n**) 30 µm.

*Sporidesmium tropicale* M.B. Ellis, Mycol. Pap. 70: 58 (1958) (Figure 11).

*Saprobic* on submerged decaying wood. **Sexual morph:** undetermined and for **asexual morph**, colonies on superficial substratum, scattered, hairy, effuse and brown to dark brown. *Mycelium* mostly immersed, composed of septate, branched, pale mid-brown to brown and with smooth-walled hyphae. *Conidiophores* 71–163 × 5–8 µm (x = 117 × 6.5 μm, *n* = 25), macronematous, mononematous, unbranched, cylindrical, erect, straight or slightly flexuous, single, 4–8-septate, dark brown, paler towards apex, smooth and thick-walled. *Conidiogenous cells* 13–22 × 5–6 µm (x = 17.5 × 5.5 μm, *n* = 25), monoblastic, holoblastic, terminal, integrated, percurrently proliferating, cylindrical and brown. *Conidia* 94–184 × 13–15 µm (x = 139.5 × 13.5 μm, *n* = 25), acrogenous, solitary, dry, pyriform, rostrate, obclavate, with a long and slender apex, straight or slightly curved, tapering to the apex, 3–5 μm wide and truncate at the base, dark brown, pale brown and 2–3 μm wide at the apex, 4–17-septate, thick-walled and with the proximal part usually verrucose.

Culture characteristics: Conidia germinating on PDA within 24 h, germ tubes produced from the basal and apical cell of conidia. Colonies growing on MEA, reaching 20–25 mm in two weeks at 25 °C, surface rough, with dense mycelia, dry, flat, rugose, from above dark brown, from below dark brown to black and not producing pigmentation in culture.

Material examined: Lancang River, Yunnan Province, China, on submerged decaying wood, acquired on July 2016, Z.L. Luo, S1689 (HKAS 115799), living culture, DLUCC 1689. (LSU) MZ420760, (ITS) MZ420745, (RPB2) MZ442698.

Notes: *Sporidesmium tropicale* was described by Ellis [99]. The species has a wide distribution, known from Bolivia, China, India, Ghana, Jamaica, Malaya, Nigeria, Sierra Leone, Sri Lanka, Thailand and USA [97,99,100,101]. Yang et al. [101] has provided sequence data for this species. In our phylogenetic analyses (Figure 9), our new collection clustered with two strains of *S. tropicale* (HKUCC 10838 and MFLU 17–0850) with strong support (100% ML and 1.00 PP). Morphology of our collection is almost the same as *S. tropicale* [101]. We, therefore, identified our species as *S. tropicale*.

Placement of *Sporidesmium tropicale* is still questionable. In the phylogenetic analyses of Yang et al. [101] *S. tropicale* clustered with *Bullimyces communis*, while Liu et al. [102] showed that it clustered with *Cryptadelphia groenendalensis*. Our phylogenetic analysis was similar to Yang et al. [101]. *Sporidesmium tropicale* clustered with *C. groenendalensis*, distant from Sporidesmiaceae (Figure 10). Further molecular studies are required to clarify the placement of this species.

**Figure 12:**
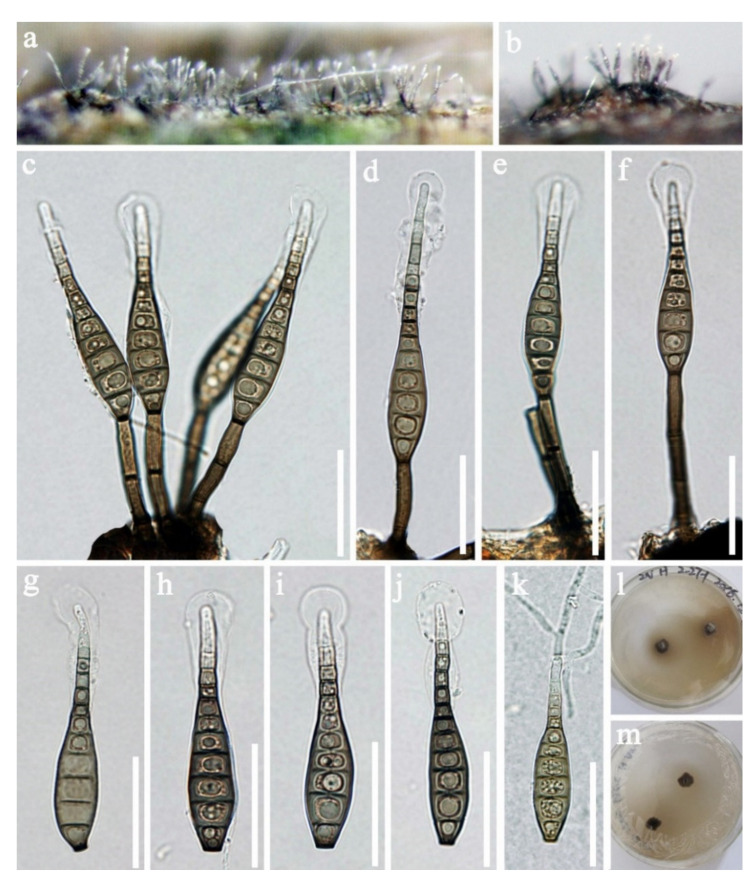
*Sporidesmium nujiangense* (HKAS 115795, holotype). (**a**,**b**) Colonies on submerged decaying wood; (**c**–**f**) Conidiophores with conidia; (**g**–**j**) Conidia; (**k**) Germinating conidium; (**l**,**m**) Culture on PDA from above and below. Scale bars: (**c**–**k**) 30 µm.

*Sporidesmium nujiangense* D.F. Bao, H.Y. Su, K.D. Hyde and Z.L. Luo, sp. nov. (Figure 12).
Index Fungorum number: IF558594; Faces of fungi number: FoF 09916
Holotype—HKAS 115795
Etymology—Referring to the place “Nujiang River” where this species was collected.

*Saprobic* on submerged decayed wood in freshwater habitats. **Sexual morph:** Undetermined. **Asexual morph:** *Colonies* effuse, velvety, hairy and brown to pale brown. *Mycelium* partly immersed, composed of septate, smooth and branched hyaline to pale brown hyphae. *Conidiophores* 31–51 × 4–5.5 µm (x = 40.8 × 4.8 μm, *n* = 27), mononematous, macronematous, erect, solitary or in small groups, cylindrical, truncate at apex, slightly swollen at the base, straight or slightly flexuous, 1–4-septate, brown to dark brown, smooth-walled. *Conidiogenous cells* 11.5–16.5 × 4–5 µm (x = 14 × 4.5 μm, *n* = 24), holoblastic, monoblastic, terminal, integrated, determinate, cylindrical and dark brown. *Conidia* 54–69 × 10–12.5 µm (x = 61.5 × 11 μm, *n* = 30), acrogenous, solitary, dry, obclavate, tapering to the apex, 2.5–3.5 µm wide at apex, truncate and 3.5–5.0 µm wide at base, brown to greyish brown, pale brown to subhyaline towards the apex, straight or slightly curved, 10–14-septate, mostly 11-septate, smooth-walled and with a mucilaginous sheath over the apex.

Culture characteristics: Conidia germinating on PDA within 24 h, with germ tubes produced from the apical cell of conidia. Colonies grew on PDA, reaching 10–15 mm in one week at 25 °C, rough surface, with dense mycelia, dry, flat, rigid, umbonate from the side view and entire edge, greyish brown from above, dark brown from below andnot producing pigmentation in culture. 

Material examined: Nujiang River, Yunnan Province, China on submerged decaying wood, in July 2016, Z.L. Luo, S983 (HKAS 115795, holotype), ex-type living culture, DLUCC 983. GenBank accession numbers: (LSU) MZ420756, (SSU) MZ420748, (ITS) MZ420741, (tef-1) MZ442691, (RPB2) MZ442695.

Notes: In the phylogenetic analyses, *Sporidesmium nujiangense* is placed as a sister taxon to *S. olivaceoconidium* with strong bootstrap support (100% ML and 1.00 BYPP, Figure 10). *Sporidesmium nujiangense* resembles *S. olivaceoconidium* in having macronematous, mononematous, septate conidiophores, holoblastic, monoblastic, integrated conidiogenous cells and solitary, obclavate, brown conidia with a mucilaginous sheath at the tip. However, *S. olivaceoconidium* has smaller conidia (25–50 × 6–10 µm) and less septa (6–10 vs. 10–14) [103]. Comparison of ITS and RPB2 regions revealed 12 bp (3.8%) and 28 bp (2.8%) differences, respectively. Therefore, we introduce *S. nujiangense* as a new species [104].

Morphologically, *Sporidesmium nujiangense* is similar to *S. appendiculatum* and *S. chiangmaiense* in having obclavate to fusiform conidia with a mucilaginous sheath over the apex. However, conidia of both *S. appendiculatum* and *S. chiangmaiense* have a filamentous appendage at the apex, while *S. nujiangense* lacks this character. Moreover, our phylogenetic analysis showed that *S. nujiangense* is placed in a different clade to *S. appendiculatum* and *S. chiangmaiense*. 

Conidia of *Sporidesmium aquaticivaginatum* and *S. guizhouense* also have a mucilaginous sheath at the conidial tip. However, *S. aquaticivaginatum* has longer conidiophores (60–125 × 4–6 μm) and smaller conidia with fewer septa (49.5–80.5 × 10.5–14 μm, 6–10-septate) [103]. *S. guizhouense* has larger conidia (46–86 × 7–11.4 µm) and more conidial septa (9–16-septate) [102].

**Figure 13:**
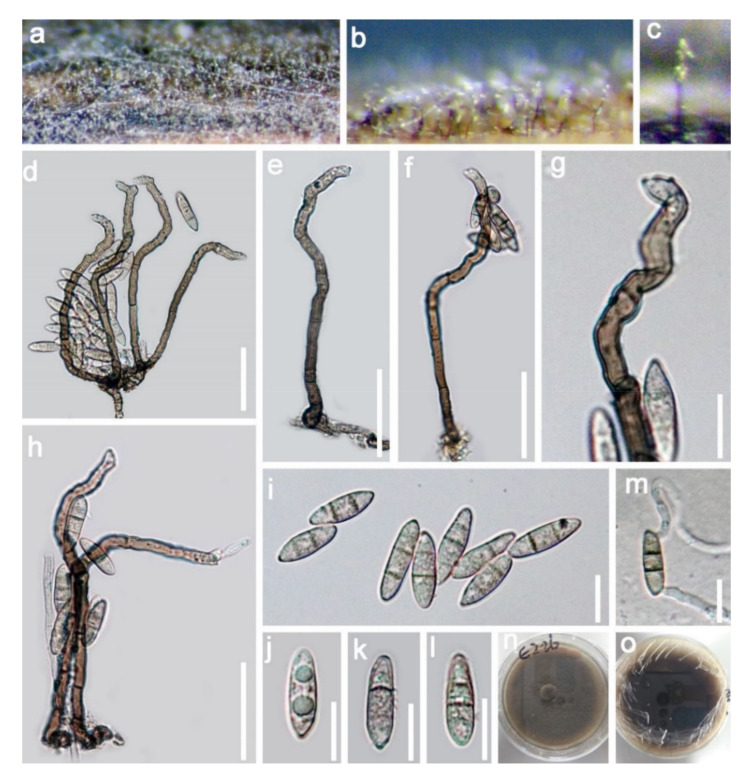
*Wongia fusiformis* (MFLU 21–0028, holotype). (**a**–**c**) Colonies on submerged decaying wood; (**d**–**f**,**h**) Conidiophores and conidia; (**g**) Conidiogenous cells; (**i**–**l**) conidia; (**m**) Germinating conidium; (**n**,**o**) Culture on PDA from above and below. Scale bars: (**d**–**f**,**h**) 30 µm; (**g**,**i**–**m**) 10 µm.

Papulosaceae Winka and O.E. Erikss., Mycoscience 41 (2):102 (2000)
*Wongia* Khemmuk, Geering and R.G. Shivas
*Wongia fusiformis* D.F. Bao, H.Y. Su, K.D. Hyde and Z.L. Luo, sp. nov. (Figure 13).
Index Fungorum number: IF558595; Facesoffungi number: FoF 09917
Holotype—MFLU 21–0028
Etymology—Referring to the fusiform conidia of this fungus

*Saprobic* on submerged decayed wood in freshwater habitats. **Sexual morph:** Undetermined. **Asexual morph:** *Colonies* on superficial substratum, hairy, scattered, granulate and black. *Mycelium* partly superficial and partly immersed, composed of branched, septate, smooth and with brown to pale brown hyphae. *Conidiophores* 70–105 × 4–5 µm, (x = 87 × 4 μm, *n* = 25) macronematous, mononematous, simple, solitary, septate, erect, straight or slightly flexuous, dark brown, paler towards apex and smooth-walled. *Conidiogenous cells* polyblastic, denticulate, terminal, sympodial and subhyaline to pale brown. *Conidia* 13–18 × 4–5 µm, (x = 15 × 5 μm, *n* = 30) acropleurogenous, solitary, fusiform, clavate, (1)–2-septate, pale brown to brown, tapering and pointed at both ends, with a guttule in each cell when young and smooth-walled.

Culture characteristics: Conidia germinating on PDA within 24 h and germ tubes produced from the basal and apical cell of conidia. Colonies grew on PDA, reaching 45–50 mm in four weeks at 25 °C, rough surface, with dense mycelia, velvety, dry, umbonate in the middle from the side view, edge undulate, brown to dark brown from above, dark brown from below and not producing pigmentation in culture.

Material examined: Sakon Nakhon, Tao Ngoi, Thailand, on submerged decaying wood, 12 November 2017, D.F. Bao, B117 (MFLU 21–0029, holotype), ex-type living culture, MFLUCC 21–0032. GenBank accession numbers: (LSU) MZ412527, (SSU) MZ413271, (ITS) MZ412515, (tef-1) MZ442689. Tao Ngoi, Sakon Nakhon, Thailand, on submerged decaying wood, 12 November 2017, D.F. Bao, B140 (MFLU 21–0028), living culture, MFLUCC 21–0028. (LSU) MZ412529, (SSU) MZ413273, (ITS) MZ412517, (tef-1) MZ442690. Lancang River, Yunnan Province, China, on submerged decaying wood, 12 November 2017, Z.L. Luo, S-1797 (HKAS 115798), living culture, DLUCC 1767. GenBank accession numbers: (LSU) MZ420761, (SSU) MZ420750, (ITS) MZ420746.

**Figure 14:**
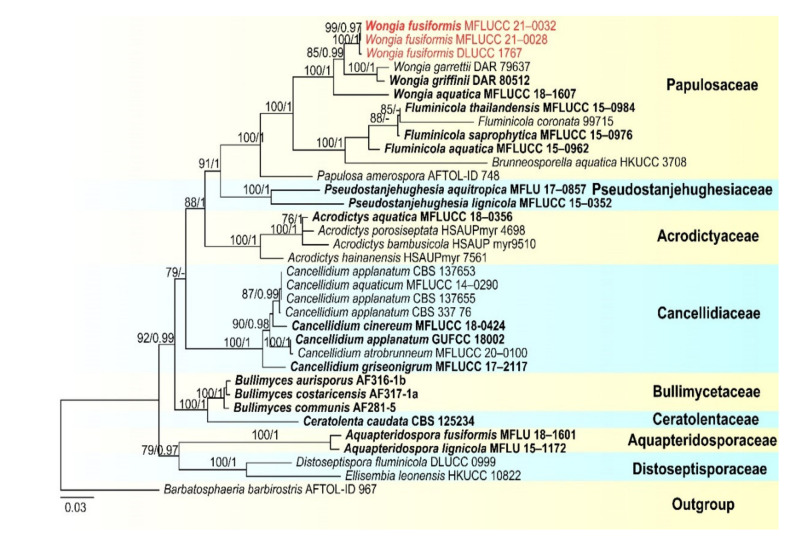
RAxML tree based on analysis of combined LSU, SSU, ITS, TEF1-a and RPB2 dataset. The combined analyses include 35 strains with 4270 characters including gaps (LSU: 1022 bp, SSU: 937 bp, ITS: 482 bp, TEF1-a: 838 bp and RPB2: 991 bp). The tree is rooted to *Barbatosphaeria barbirostris* (AFTOL-ID 967). Tree topology of the maximum likelihood analysis and Bayesian analysis are similar. The RAxML analysis of the combined dataset yielded a best scoring tree (Figure 14) with a final ML likelihood value of −21,062.963451. The matrix had 1421 distinct alignment patterns, with 47.50% undetermined characters or gaps. Estimated base frequencies were as follows: A = 0.248137, C = 0.246561, G = 0.278073, T = 0.227229; substitution rates AC = 1.201394, AG = 2.386577, AT = 1.207334, CG = 1.204705, CT = 5.750613, GT = 1.000000; gamma distribution shape parameter α = 0.224853. Bootstrap values for maximum likelihood (ML) ≥ 75% and Bayesian posterior probabilities (PP) ≥ 0.95 are labelled on the nodes. The ex-type strains are in bold and black. The newly obtained sequences are indicated in red.

Notes: in the current phylogenetic analyses, our three new isolates clustered together, sister to the clade of *Wongia garrettii* and *W. griffinii* with strong bootstrap support (100 ML/1.00 PP, Figure 14). *Wongia garrettii* and *W. griffinii* were introduced with a sexual morph [105], but *W. fusiformis* is known only from the asexual morph. Thus, we are unable to compare their morphology but, they are phylogenetically distinct (Figure 14). *Wongia fusiformis* resembles *W. aquatica* in having macronematous, mononematous, solitary, unbranched conidiophores, polyblastic, sympodial, denticulate conidiogenous cells and acropleurogenous, solitary and fusiform conidia [28]. However, *W. fusiformis* differs from *W. aquatica* by the color (pale brown to brown vs. dark brown at the two central cells and paler at end cells), shape (tapering and pointed at both ends vs. rounded and narrow at apex and truncate at base) and septation (1–2-septate vs. 3-septate) of conidia. Phylogenetic analyses also showed that they formed different clades in *Wongia*; therefore, we identified our isolate as a new species.

**Figure 15:**
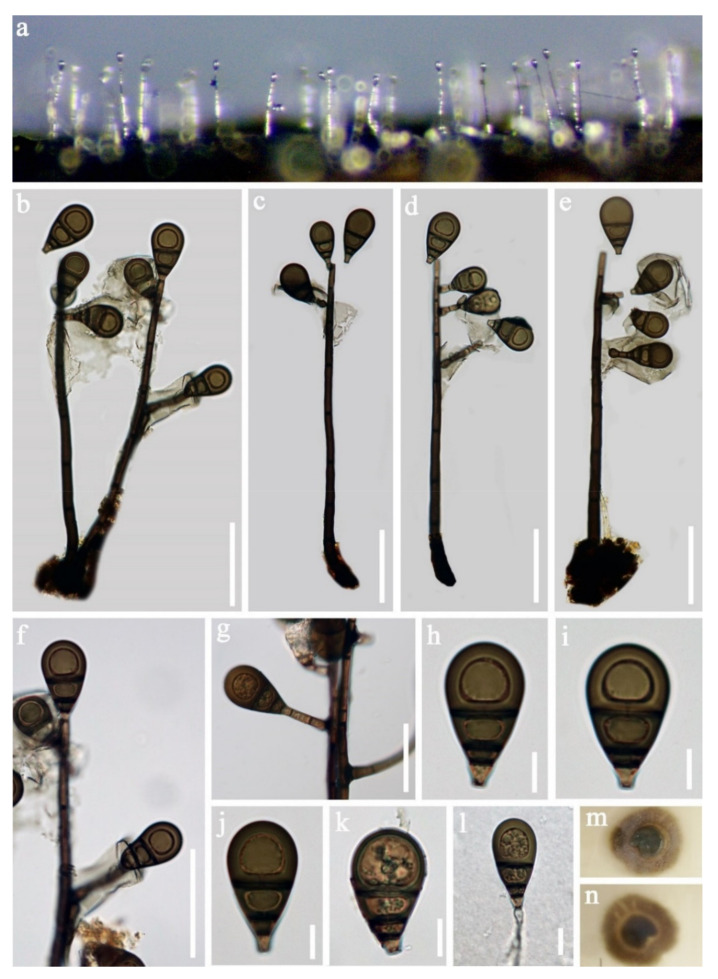
*Neospadicoides thailandica* (MFLU 21–0032, holotype). (**a**) Colonies on submerged decaying wood; (**b**–**e**) Conidiophores and conidia; (**f**,**g**) Conidiogenous cells with conidia; (**h**–**k**) conidia; (**l**) Germinating conidium; (**m**,**n**) Culture on PDA from above and below. Scale bars: (**b**–**f**) 50 µm; (**g**) 30 µm; (**h**–**l**) 10 µm.

Xenospadicoidales Hern.-Restr., J. Mena and Gené
Xenospadicoidaceae Hern.-Restr., J. Mena and Gené
*Neospadicoides thailandica* D.F. Bao, H.Y. Su, K.D. Hyde and Z.L. Luo, sp. nov. (Figure 15).
Index Fungorum number: IF 558596; Faces of fungi number: FoF 09918
Holotype: MFLU 21–0032
Etymology: Referring to the species was collected from Thailand.

*Saprobic* on submerged decayed wood in freshwater habitats. **Sexual morph:** Undetermined. **Asexual morph:** *Colonies* on superficial substratum, scattered, granulate and dark brown to black. *Mycelium* partly superficial and partly immersed, composed of branched, septate, brown to dark brown and branched hyphae. *Conidiophores* 165–235 × 5–7 μm (x = 200 × 6 μm, *n* = 15), macronematous, mononematous, erect, solitary, cylindrical, straight or flexuous, dark brown to black, smooth, with thick, dark brown to black septa, with 2–4-branches in the middle to upper part, branches 15–35 × 4–6 μm (x =24 × 5 μm, *n* = 15), 1–3-septate, medium to dark brown, with a hyaline to brown sheath over the branches. *Conidiogenous cells* holoblastic, monoblastic, integrated, terminal and dark brown. *Conidia* 29–35 µm long, 15–20 µm wide at apex, 5–8 µm wide at base (*n* = 30), acrogenous or acropleurogenous, solitary, obovoid, 3-septate, brown to dark brown, rounded at the apex, truncate at the base, thick- and smooth-walled and sometimes with a subhyaline to brown sheath.

Culture characteristics: Conidia germinated on PDA within 24 h and germ tubes produced from the basal cell of conidia. Colonies growing on PDA, reached 20–25 mm in two weeks at 25 °C, rough surface, dense, circular, slightly raised to umbonate with the entire edge, floccose, brown from above, dark brown from below and not producing pigmentation in culture. 

Material examined: Sakon Nakhon, Tao Ngoi, Thailand, on submerged decaying wood, 12 November 2017, D.F. Bao, B159 (MFLU 21–0032, holotype), ex-type living culture, MFLUCC 21–0031. GenBank accession numbers: (LSU) MZ412532, (SSU) MZ413275, (ITS) MZ412520. Thailand, Tao Ngoi, Sakon Nakhon, on submerged decaying wood, 12 November 2017, D.F. Bao, B168 (MFLU 21–0031), living culture, MFLUCC 21–0029. GenBank accession numbers: (LSU) MZ412533, (SSU) MZ413276, (ITS) MZ412521.

**Figure 16:**
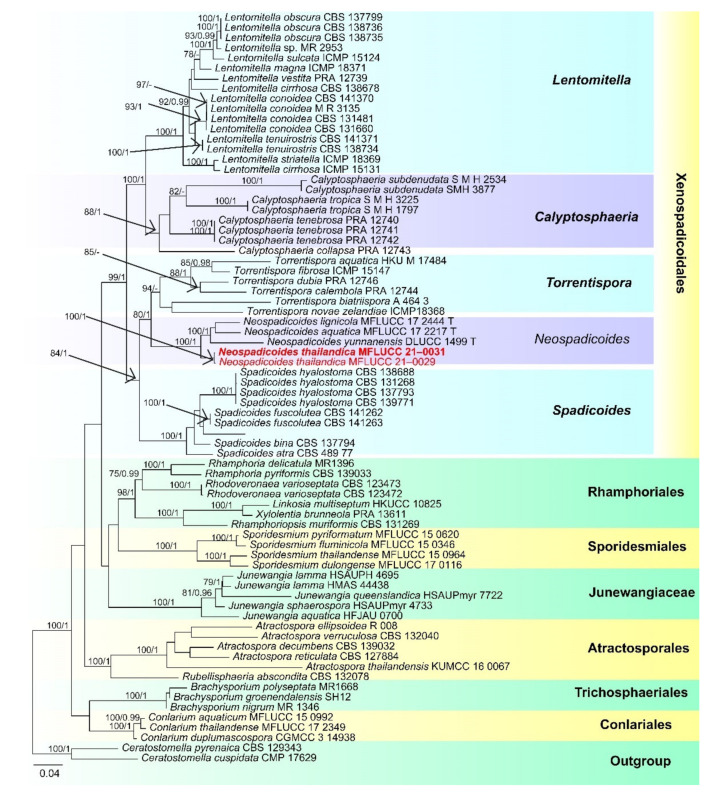
RAxML tree based on analysis of combined LSU, SSU, ITS and RPB2 dataset. The combined analyses include 74 strains with 4212 characters including gaps (LSU: 1136 bp, SSU: 1139 bp, ITS: 564 bp and RPB2: 1144 bp). The tree is rooted to *Ceratostomella pyrenaica* (CBS 129343) and *C. cuspidata* (CMP 17629). Tree topology of the maximum likelihood analysis and Bayesian analysis are similar. The RAxML analysis of the combined dataset yielded a best scoring tree (Figure 16) with a final ML likelihood value of −39,690.726824. The matrix had 1778 distinct alignment patterns, with 30.92% undetermined characters or gaps. Estimated base frequencies were as follows: A = 0.248100, C = 0.243558, G = 0.284201, T = 0.224142; substitution rates AC = 1.409308, AG = 3.086772, AT = 1.346045, CG = 1.235614, CT = 5.843915, GT = 1.000000; gamma distribution shape parameter α = 0.200153. Bootstrap values for maximum likelihood (ML) ≥ 75% and Bayesian posterior probabilities (PP) ≥ 0.95 are labelled on the nodes. The newly obtained sequence is indicated in red.

Notes: *Neospadicoides* was introduced by Luo et al. [28] with three new species: *N. lignicola*, *N. aquatica* and *N. yunnanensis*. The genus is characterized by macronematous, mononematous, septate conidiophores, holoblastic, terminal, integrated conidiogenous cells and acrogenous or acropleurogenous, fusiform, obovoid and septate conidia. *Neospadicoides thailandica* fits well with the generic concept. However, *N. thailandica* is unique in the genus by its branched conidiophores, sometimes with a hyaline to brown sheath over the branches and conidia. Other species in the genus lack a sheath and the conidiophores are unbranched. Phylogenetic analyses showed that our collections formed a distinct and stable lineage within *Neospadicoides* (Figure 16). Therefore, we introduce our collections as a new species. All species in the genus were reported from freshwater habitats in China [28], while *N. thailandica* was collected from Thailand.

**Figure 17:**
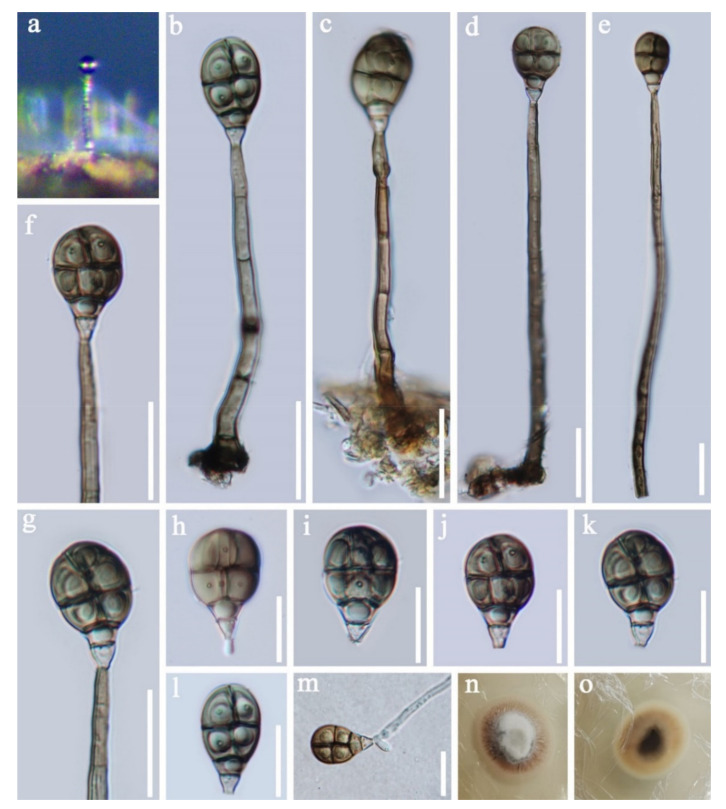
*Acrodictys liputii* ((MFLU 21–0034)) (**a**) Colony on submerged decaying wood; (**b**–**e**) Conidiophores with conidia; (**f**,**g**) Conidiogenous cells with conidia; (**h**–**l**) Conidia; (**m**) Germinating conidium; (**n**,**o**) Culture on PDA from above and below. Scale bars: (**b**–**g**) 20 µm; (**h**–**m**) 15 µm.

Acrodictyaceae J.W. Xia and X.G. Zhang
*Acrodictys* M.B. Ellis
*Acrodictys liputii* L. Cai, K.Q. Zhang, McKenzie, W.H. Ho and K.D. Hyde, Nova Hedwigia 75 (3–4): 526, 2002. (Figure 17).

*Saprobic* on submerged decaying wood. **Sexual morph:** Undetermined. **Asexual morph:** *Colonies* on superficial substratum, effuse, scattered, hairy and brown to dark brown. *Mycelium* partly superficial and partly immersed, composed of branched, septate, brown to dark brown and with smooth hyphae. *Conidiophores* 97–180 × 4.5–6 µm (x = 138.5 × 5.5 μm, *n* = 20), macronematous, mononematous, simple, cylindrical, erect, 4–8-septate, straight or slightly flexuous, pale brown to brown and smooth-walled. *Conidiogenous cells* holoblastic, monoblastic, terminal, integrated, cylindrical and truncate, brown. *Conidia* 11.5–15 × 3.4–5 µm (x = 13 × 4 μm, *n* = 30), acrogenous, dry, solitary, muriform, subglobose, obovoid to pyriform, truncated at base, rounded at apex, pale brown to subhyaline at the basal cell, brown to greyish brown at other parts, with 3–4- transverse septa and 2- longitudinal septa, constricted at the septa, with conspicuous pores in the septa and smooth.

Culture characteristics: Conidia germinating on PDA within 24 h, germ tubes produced from the basal cell of conidia. Colonies grew on PDA, reaching 20–25 mm in two weeks at 25 °C, surface rough, circular, with dense mycelia, velvety, slightly raised to umbonate, with edge entire, floccose, white at center from above, yellow at the edge, dark brown at center from below, pale yellow to greyish yellow at the edge and not producing pigmentation in culture. 

Material examined: Phra Khanong Nuea, Bangkok Province, Thailand, on submerged decaying wood, 1 October 2017, Z.L. Luo, B-50 (MFLU 21-0034), ex-type living culture MFLUCC 18–0323. GenBank accession numbers: (LSU) MZ412524, (SSU) MZ413269, (ITS) MZ412512.

**Figure 18:**
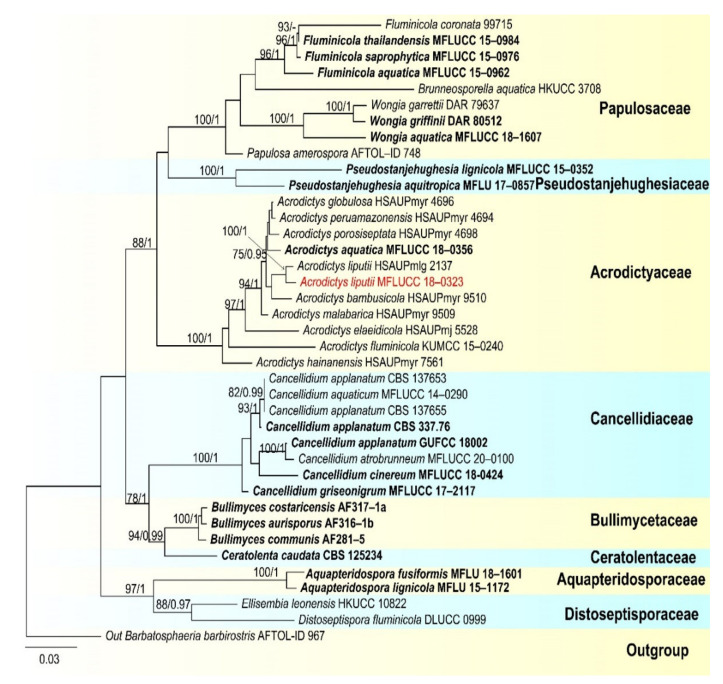
RAxML tree based on analysis of combined LSU, ITS and SSU dataset. The combined analyses include 39 strains with 2797 characters including gaps (LSU: 839 bp, ITS: 595 bp and SSU: 1363 bp). The tree is rooted to *Ceratostomella pyrenaica* (CBS 129343) and *C. cuspidate* (CMP 17629). Tree topology of the maximum likelihood analysis and Bayesian analysis are similar. The RAxML analysis of the combined dataset yielded a best scoring tree (Figure 18) with a final ML likelihood value of −12,848.718654. The matrix had 829 distinct alignment patterns, with 39.95% undetermined characters or gaps. Estimated base frequencies were as follows: A = 0.250988, C = 0.227781, G = 0.281648, T = 0.239583; substitution rates AC = 1.292145, AG = 1.860337, AT = 1.456924, CG = 0.675369, CT = 5.012007, GT = 1.000000; gamma distribution shape parameter α = 0.207257. Bootstrap values for maximum likelihood (ML) ≥ 75% and Bayesian posterior probabilities (PP) ≥ 0.95 are labelled on the nodes. The ex-type strains are in bold and black. The newly obtained sequence is indicated in red.

Notes: In the phylogenetic analyses, our isolate clustered with *Acrodictys liputii* (HSAUPmlg 2137) with strong bootstrap support (100% ML/1.00 PP, Figure 18). Morphology of our isolate and the holotype of *A. liputii* are indistinguishable. *Acrodictys liputii* was introduced by Cai et al. [106] and collected on submerged bamboo culm from the Philippines. Xia et al. [107] provided sequence data for this species and first reported this species from China. Our collection is the first record from Thailand.

**Figure 19:**
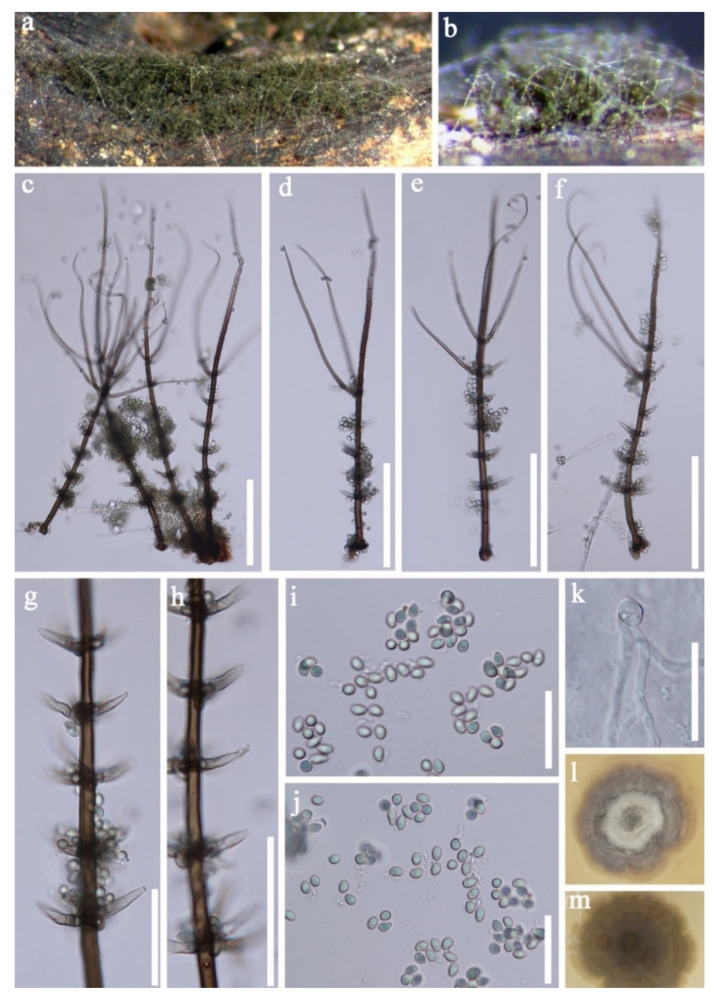
*Chloridium gonytrichii* (MFLU 21–0026). (**a**,**b**) Colonies on submerged decaying wood; (**c**–**f**) Conidiophores with conidia; (**g**,**h**) Conidiogenous cells with conidia; (**i**,**j**) Conidia; (**k**) Germinating conidia; (**l**,**m**) Culture on PDA from surface and reverse. Scale bars: (**c**–**f**) 80 µm; (**g**,**h**) 30 µm; (**j**,**k**) 20 µm.

Chaetosphaeriaceae Réblová, M.E. Barr and Samuels, Sydowia 51(1): 56 (1999)
*Chloridium* Link, Mag. Gesell. naturf. Freunde, Berlin 3(1–2): 13 (1809)
*Chloridium gonytrichii* (F.A. Fernández and Huhndorf) Réblová & Seifert, in Réblová et al., IMA Fungus 7(1): 134 (2016) (Figure 19).
≡ *Gonytrichum macrocladum* (Sacc.) S. Hughes, Trans. Br. Mycol. Soc. 34(4): 565 (1952) (1951)
≡ *Chloridium aseptatum* M.J. Wei and H. Zhang, in Wei, Zhang, Dong, Boonmee & Zhang, Phytotaxa 362(2): 191 (2018)
≡ *Chloridium macrocladum* (Sacc.) Karun, Maharachch., C.H.Kuo and K.D.Hyde, in Yuan et al., Fungal Diversity [74] (2020)

*Saprobic* on decaying wood submerged in freshwater habitats. **Sexual morph:** Undetermined. **Asexual morph:** *Colonies* effuse, hairy and dark green to dark brown. *Mycelium* superficial. *Conidiophores* 190–336 × 4.5–6.5 µm, (x = 262.5 × 5.5 μm, *n* = 15), macronematous, mononematous, single, unbranched, septate, gradually becoming narrower towards the apex, with 3–4 long branches at the upper part, with 2–5 whorls of phialides in the midsection to lower section and a single phialide at the apex, dark brown and paler towards the apex. *Conidiogenous cells* 9–16.5 × 3–4 µm, (x = 12.5 × 3.5 μm, *n* = 25), cylindrical to lageniform, phialides, producing conidia from multiple entero-blastic conidiogenous loci and phialides borne on collar hyphae around the conidiophore. *Conidia* 3.5–4.5 × 2.5–3.0 µm, (x = 4 × 2.5 μm, *n* = 35), globose to subglobose, aseptate and hyaline to subhyaline. 

Culture characteristics: Conidia germinated on PDA within 24 h. Colonies grew on PDA, reaching 20–25 mm in two weeks at 25 °C, with rough surface and dense mycelia, dry, rigid, umbonate from the side view and entire edge, greyish brown to pale brown at the margin from above, white to grey at the center, dark grey to brown from below and not producing pigmentation in culture.

Material examined: Sakon Nakhon, Tao Ngoi, Thailand, saprobic on submerged decaying wood, on 12 November 2018, D.F. Bao, B130 (MFLU 21–0026), living culture, MFLUCC 21–0026. GenBank accession numbers: (LSU) MZ412528, (SSU) MZ413272, (ITS) MZ412516. Khwaeng Phra Khanong Nuea, Khet Watthana Krung Thep Maha Nakhon, Thailand, saprobic on decaying wood submerged in a freshwater stream was acquired on 1 October 2017, Z.L. Luo, B-47 (MLU 21–0035, holotype), living culture MFLUCC 18–0251. GenBank accession numbers: (LSU) MZ412523, (ITS) MZ412511.

Notes: our new collection is identified as *Chloridium gonytrichii* based on both phylogeny and morphology. *Chloridium gonytrichii* is characterized by single, unbranched conidiophores with 5–8 whorls of phialides in the midsection, a single phialide at the apex, phialide and cylindrical to lageniform conidiogenous cells ellipsoid conidia [28,108]. Our collection is almost the same as the holotype of *C. gonytrichii* [108]. Therefore, we identified our new collection as *C. gonytrichii*.

**Figure 20:**
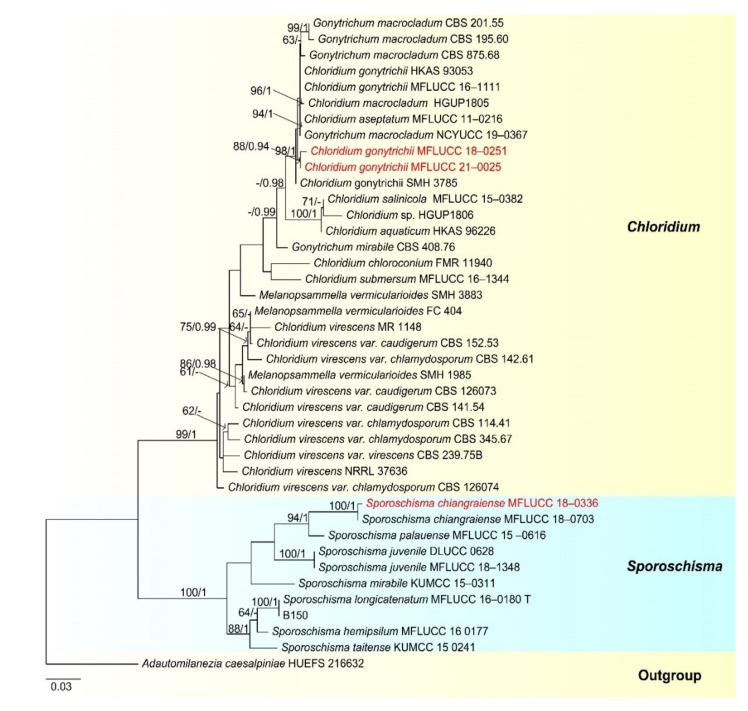
RAxML tree based on analysis of combined LSU and ITS dataset. The combined analyses include 41 strains with 1575 characters including gaps (LSU: 976 bp and ITS: 599 bp). The tree is rooted to *Adautomilanezia caesalpiniae* (HUEFS 216632). Tree topology of the maximum likelihood analysis and Bayesian analysis are similar. The RAxML analysis of the combined dataset yielded a best scoring tree with a final ML likelihood value of –6385.584083. The matrix had 519 distinct alignment patterns, with 20.19% undetermined characters or gaps. Estimated base frequencies were as follows: A = 0.234371, C = 0.252713, G = 0.308937, T = 0.203978; substitution rates AC = 2.166481, AG = 2.769451, AT = 2.423722, CG = 1.278303, CT = 9.483184, GT = 1.000000; gamma distribution shape parameter α = 0.168757. Bootstrap values for maximum likelihood (ML) ≥ 60% and Bayesian posterior probabilities (PP) ≥ 0.85 are labelled on the nodes. The newly obtained sequences are indicated in red.

*Chloridium gonytrichii* was described by Fernández et al. [108] with its sexual and asexual morphs. It was originally placed in *Melanopsammella*. Crous et al. [109] provided sequence data for this species and phylogenetic analysis showed that *C. gonytrichii* clustered with *Melanopsammella vermicularioides*. Réblová et al. [110] transferred it to *Chloridium*, synonymized *C. gonytrichii* under *Melanopsammella gonytrichii*. Phylogenetic analyses of Luo et al. [28] and Lin et al. [111] showed that *gonytrichii* formed a stable and well-supported lineage within *Chloridium*. In our analyses, *C. aseptatum* (MFLUCC 11–0216), four strains of *C. macrocladum* (CBS 201.55, CBS 195.60, CBS 875.68 and NCYUCC 19–0367) and five strains of *C. gonytrichii* (MFLUCC 16–1111, HKAS:93053, SMH 3785, MFLUCC 18–0251, MFLUCC 21–0025) clustered together in a distinct clade within *Chloridium* with strong bootstrap support (98 ML/1.00 PP). Both morphology and phylogeny of *C. macrocladum* is indistinguishable to *C. gonytrichii* (Figure 20) [28,108,112], thus we propose *C. macrocladum* as a synonym of *C. gonytrichii*. 

*Chloridium aseptatum* was introduced by Wei et al. [113], based on morphology and ITS sequence data, Yuan et al. [112] synonymized *C. macrocladum* instead *C. aseptatum*, our phylogenetic analysis also obtained the same result, *C. aseptatum* (MFLUCC 11–0216) clustered with *C. gonytrichii* (Figure 20). Hence, we are in agreement with Yuan et al. [112], and synonymized both *C. macrocladum* and *C. aseptatum* under *C. gonytrichii*, based on phylogeny and morphology.

**Figure 21:**
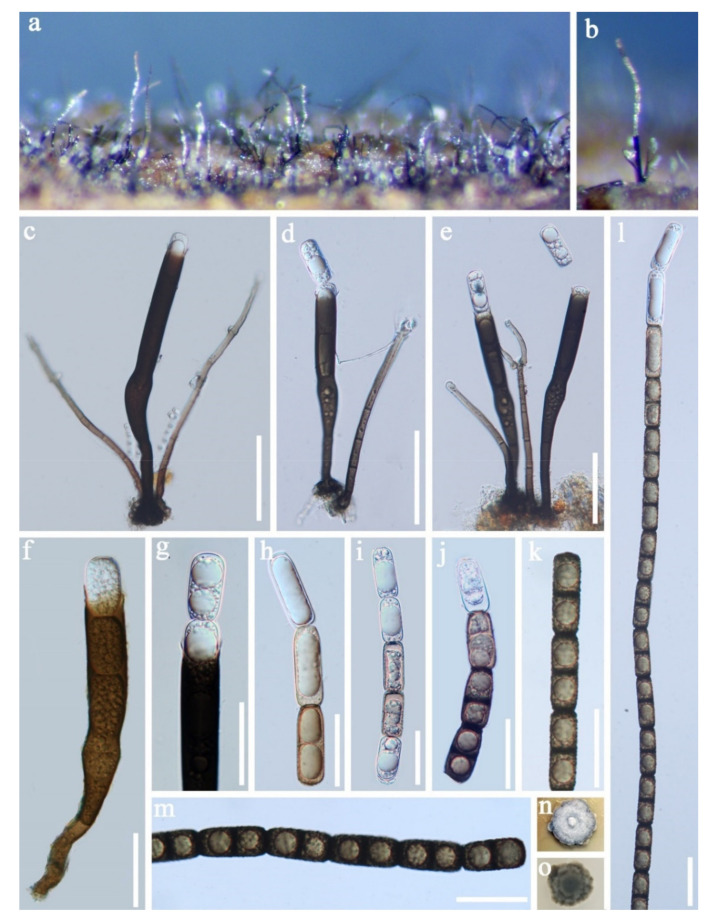
*Sporoschisma chiangraiense* (MFLU 21–0036) (**a**,**b**) Colonies on submerged decaying wood; (**c**–**e**) Conidia and conidiophores with setae; (**f**) Conidiophore with conidia; (**g**) Conidiogenous cells with conidia; (**h**–**m**) conidia; (**n**,**o**) Culture on MEA from surface and reverse. Scale bars: (**c**–**f**) 50 µm; (**g**–**m**) 30 µm.

*Sporoschisma* Berk. & Broome
*Sporoschisma chiangraiense* N.G. Liu and K.D. Hyde, Fungal Diversity 96: 160 (2019) (Figure 21).

*Saprobic* on decaying wood submerged in freshwater habitats. **Sexual morph:** Undetermined. **Asexual morph**: *Colonies* were effuse, hairy and black, with long chains of conidia. *Mycelium* immersed, composed of brown to dark brown and unbranched hyphae. *Setae* 99−162 × 4–6 µm, capitate, scattered or in groups mixed with conidiophores, straight or slightly flexuous, apex swollen, surrounded by hyaline mucilage, median brown, subhyaline to pale brown near the apex and septate. *Conidiophores* 120–170 µm long, 12–16 µm wide at venter, 5–8 µm wide below venter, 13–15 µm wide above, macronematous, mononematous, solitary or in a small group of 2–3, erect, unbranched, straight or slightly flexuous, arising from dark brown to black bulbous base, dark brown to black, paler at the torn apex, composed of a cylindrical stipe and a swollen venter with a long cylindrical neck, sometimes proliferating percurrently and smooth. *Conidiogenous cells* monophialidic, integrated, percurrent, lageniform, brown and frayed at the apex. *Conidia* 27–32 × 11–13 µm (x = 29 × 12 µm, *n* = 30), catenate, cylindrical, hyaline and smooth-walled when young, becoming olivaceous brown to dark brown and verrucose at maturity, with a big guttule in each cell when mature, 1-septate, conspicuously darkened and slightly constricted at the septa and darkened at both ends.

Culture characteristics: Conidia germinated on PDA within 24 h and colonies grew on MEA, reaching 15–25 mm in two weeks at 25 °C, withrough surface and dense mycelia, velvety, dry, umbonate in the middle from the side view, edge undulate, white to pale grey from above, dark grey to brown at the margin from below andnot producing pigmentation in culture.

Material examined: Phra Khanong Nuea, Bangkok Province, Thailand, on submerged decaying wood, 1 October 2017, Z.L. Luo, B81 (MFLU 21–0036, holotype), ex-type living culture MFLUCC 18–0336. GenBank accession numbers: (LSU) MZ412525, (ITS) MZ412513.

Notes: *Sporoschisma chiangraiense* was introduced by Hyde et al. [90], it is unique in *Sporoschisma* by having 1-septate, verrucose conidia, while conidia of other species are multi-septate with smooth-walled. Phylogenetic analyses showed that our new isolate MFLUCC 18–0336 clustered with the ex-type strain of *S. chiangraiense* (MFLUCC 18–0703) with high bootstrap support (Figure 19). Morphology of our isolate and holotype of *S. chiangraiense* are almost the same, except the wall of conidia; conidia of our isolate are verrucose at maturity, while this character was not observed in holotype. Based on both phylogenetic analyses and morphology characters, we identified our new isolate as *S. chiangraiense*.

**Figure 22:**
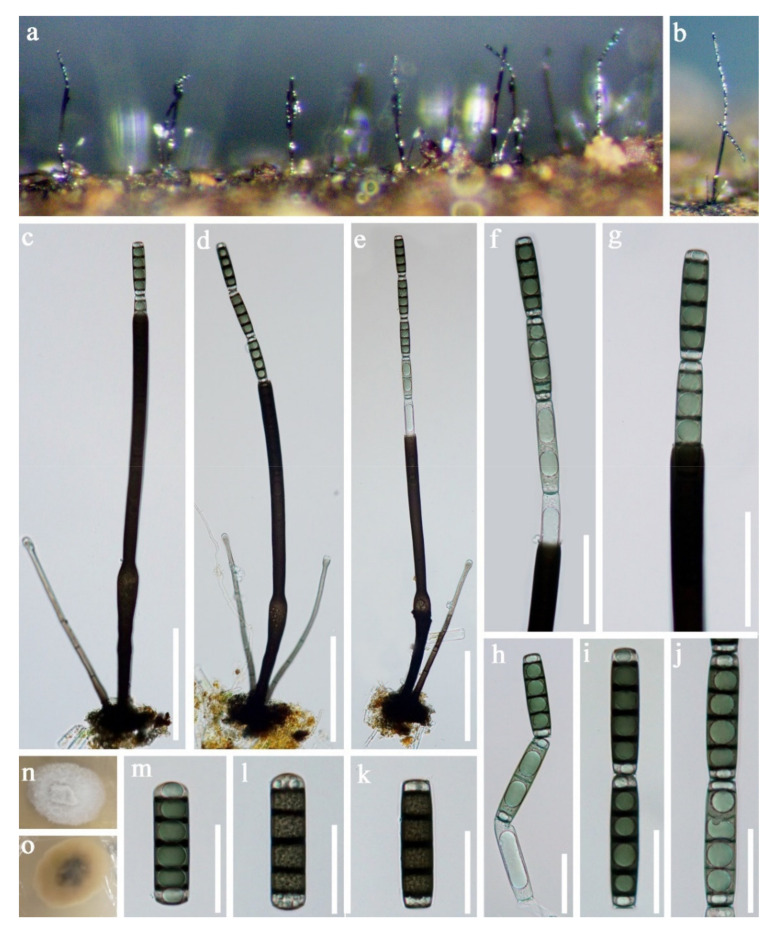
*Sporoschisma longicatenatum* (MFLU 21–0033) (**a**,**b**) Colonies on submerged decaying wood; (**c**–**e**) Conidia and conidiophores with setae; (**f**,**g**) Conidiogenous cells with conidia; (**h**–**m**) conidia; (**n**,**o**) Culture on PDA from surface and reverse. Scale bars: (**c**–**e**) 100 µm; (**f**,**g**) 50 µm; (**h**–**m**) 30 µm.

*Sporoschisma longicatenatum* Jing Yang, Jian K. Liu and K.D. Hyde, in Yang et al., Phytotaxa 289(2): 152 (2016) (Figure 22).

*Saprobic* on decaying wood submerged in freshwater habitats. **Sexual morph:** Undetermined. **Asexual morph:** *Colonies* effuse, hairy, black and with long chains of conidia. *Mycelium* immersed, composed of brown to dark brown, branched and with smooth hyphae. *Setae* 143−177 × 5–7 μm, capitate, simple or in groups, cylindrical, swollen at apex, pale to medium brown, paler towards apex, straight or slightly flexuous, 3−5-septate and smooth. *Conidiophores* macronematous, mononematous, solitary with 0−4 setae, erect, straight or slightly flexuous, smooth, dark brown to black, arising from dark brown to black bulbous base, composed of a cylindrical stipe and a swollen venter with a long cylindrical neck, 315–365 μm long, 11–13 μm wide below venter and 12–14 μm wide above, 15–22 μm wide at venter. *Conidiogenous cells* monophialidic, terminal, integrated, determinate, lageniform, dark brown to black and frayed at the apex. *Conidia* 43–48 × 9–12 μm (x = 45 × 11 μm, *n* = 35), catenate, cylindrical to doliiform, rounded at both ends, mostly 5-euseptate, conspicuously darkened at the septa, hyaline when young, dark olive green to brown at maturity, end cells were hyaline to pale brown and shorter than the inner cells, with guttules in each cell. 

Culture characteristics: Conidia germinated on PDA within 24 h and colonies grew on PDA, reaching 20–25 mm in two weeks at 25 °C, with rough surface and dense mycelia, dry, velvety, raised from the side view and entire edge, white to pale grey from above; greyish white from below and not producing pigmentation in culture.

Material examined: Sakon Nakhon, Tao Ngoi, Thailand, on submerged decaying wood, 12 November 2017, D.F. Bao, B150 (MFLU 21–0033), living culture, MFLUCC 21–0033. GenBank accession numbers: (LSU) MZ412531, (ITS) MZ412519.

Notes: Morphology of our new collection is indistinguishable with *Sporoschisma longicatenatum***.** Phylogenetic analyses showed that our new collection clustered with the ex-type strain of *S. longicatenatum* with high bootstrap (Figure 20), we therefore identified our collection as *S. longicatenatum*. *Sporoschisma longicatenatum* was introduced by Yang et al. [28] from freshwater habitat in Thailand and this species were exclusively reported from freshwater habitats and only known from Thailand.

**Figure 23:**
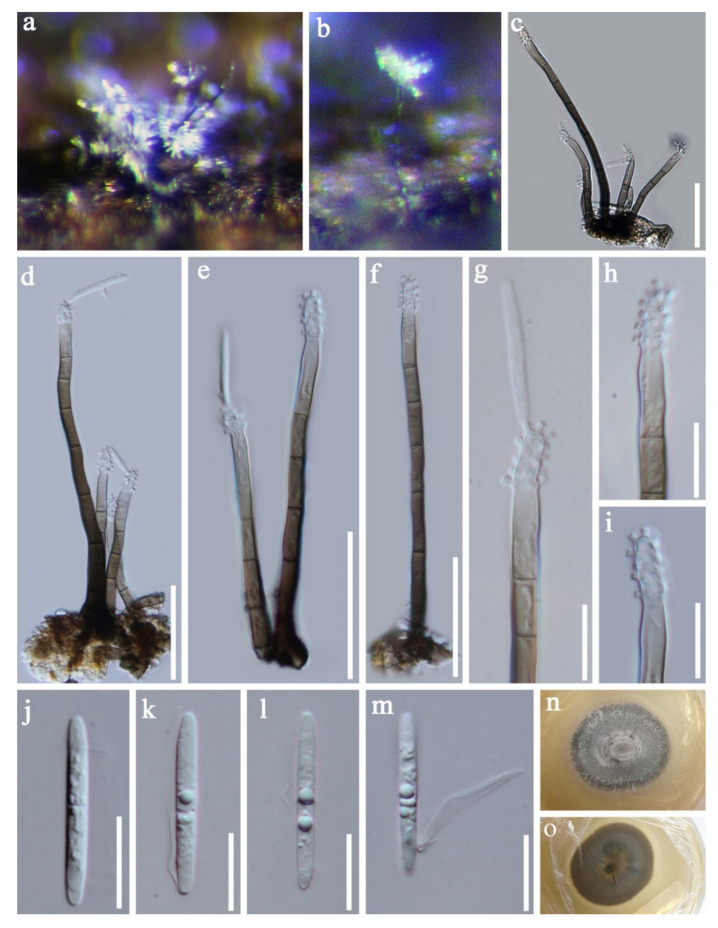
*Pseudodactylaria aquatica* (MFLU 21–0037, holotype). (**a**,**b**) Colonies on submerged decaying wood; (**c**–**f**) Conidiophores, conidiogenous cells with denticles and conidia; (**g**–**i**) Conidiogenous cells with denticles; (**j**–**m**) Conidia; (**n**,**o**) Culture on MEA from surface and reverse. Scale bars: (**c**–**f**) 30 µm; (**g**–**m**) 10 µm.

Pseudodactylariales Crous,
Pseudodactylariaceae Crous
Pseudodactylaria Crous
*Pseudodactylaria aquatica* D.F. Bao, H.Y. Su, K.D. Hyde and Z.L. Luo, sp. nov. (Figure 23).
Index Fungorum number: IF 558597; Faces of fungi number: FoF 09919
Holotype—MFLU 21–0037
Etymology—Referring the fungus was collected form aquatic habitat.

*Saprobic* on decaying wood submerged in freshwater habitats. **Sexual morph:** Undetermined. **Asexual morph:** *Colonies* effuse, shining, greyish brown, velvety and in groups. *Mycelium* partly immersed, composed of septate, brown, branched and with smooth hyphae. *Conidiophores* 40–100 × 3.5–4.5 µm, (x = 70 × 4.0 μm, *n* = 30), macronematous, mononematous, erect, unbranched, 3–6-septate, straight or slightly flexuous, brown to dark brown, paler towards the apex, often in a small group of 3–5 and smooth-walled. *Conidiogenous cells* 11.5–22 × 3.0–4.5 µm, (x = 11.5 × 3.0 μm, *n* = 30), polyblastic, denticulate, integrated, cylindrical and hyaline. *Conidia* 20–23 × 2.5–3.5 µm, (x = 21.5 × 3.0 μm, *n* = 35), acrogenous, solitary, cylindrical, narrowly fusiform, straight, rounded at the both bends, 0-1-septate, guttules, hyaline and smooth-walled, with a hyaline appendage at the base. 

Culture characteristics: Conidia germinated on PDA within 24 h and colonies grew on MEA, reaching 25–30 mm in three weeks at 25 °C, with rough surface and dense mycelia, dry, raised from the side view and entire edge, dark grey at the margin from above, pale grey at the middle, dark brown at the margin from below, greyish brown at the middle and not producing pigmentation in culture. 

Material examined: Khok Pho District, Thailand, on submerged decaying wood, 28 August 2017, C.G. Lin, 1F–8–1. B6 (MFLU 21–0037, holotype), ex-type living culture, MFLUCC 18–0201. GenBank accession numbers: (LSU) MZ412522, (ITS) MZ412510.

**Figure 24:**
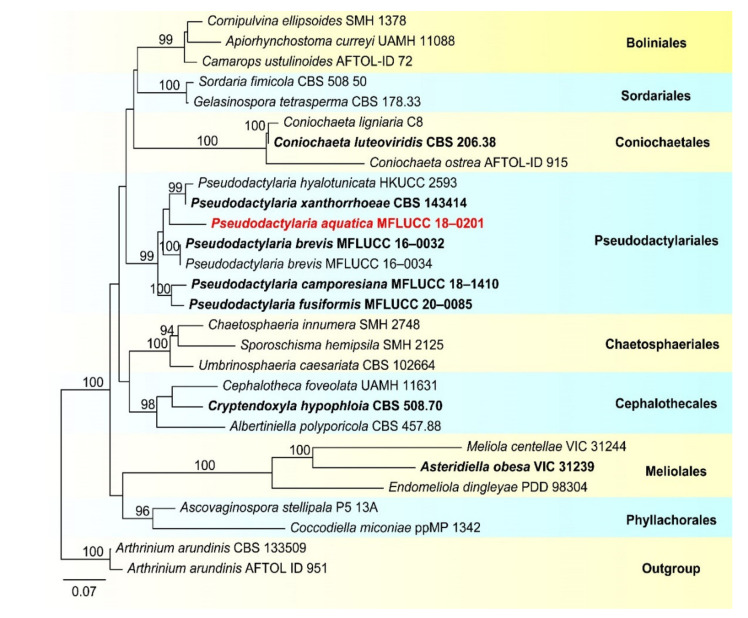
RAxML tree based on analysis of combined LSU and ITS dataset. The combined analyses include 28 strains with 1558 characters including gaps (LSU: 932 bp and ITS: 626 bp). The tree is rooted to *Arthrinium arundinis* (CBS 133509 and AFTOL-ID 951). Tree topology of the maximum likelihood analysis and Bayesian analysis are similar. The RAxML analysis of the combined dataset yielded a best scoring tree (Figure 24) with a final ML likelihood value of −11,302.425391. The matrix had 843 distinct alignment patterns, with 29.21% undetermined characters or gaps. Estimated base frequencies were as follows: A = 0.249344, C = 0.235680, G = 0.296458, T = 0.218518; substitution rates AC = 1.060678, AG = 2.194343, AT = 1.846441, CG = 0.792078, CT = 6.240676, GT = 1.000000; gamma distribution shape parameter α = 0.380855. Bootstrap values for maximum likelihood (ML) equal to or ≥75% and clade credibility values ≥0.95 from Bayesian-inference analysis labelled on the nodes. The ex-type strains are in bold and black. The newly obtained sequence is indicated in red.

Notes: Species of *Pseudodactylaria* are characterized by single, unbranched, septate, hyaline conidiophores, polyblastic, denticulate conidiogenous cells and solitary, fusoid-ellipsoid, hyaline conidia [69,114,115]. *Pseudodactylaria aquatica* fits well with the generic concept of *Pseudodactylaria*, such as, single, unbranched, septate conidiophores, polyblastic, denticulate conidiogenous cells and solitary, fusoid-ellipsoid and hyaline conidia. However, our species can be distinguished from other *Pseudodactylaria* species in having brown to dark brown conidiophores, which are in groups of 3–5, and cylindrical, narrowly fusiform conidia with a hyaline appendage at the base. While other *Pseudodactylaria* species have single conidiophores and conidia are lacking an appendage. Phylogenetic analysis showed that *P. aquatica* formed a distinct lineage within the genus (Figure 24) [69,114,116].

*Pseudodactylaria* species were reported as saprobes from freshwater or terrestrial habitats in Australia [116], China [55,69] and Thailand [114]. Our new species was collected from freshwater habitats in Thailand.

**Figure 25:**
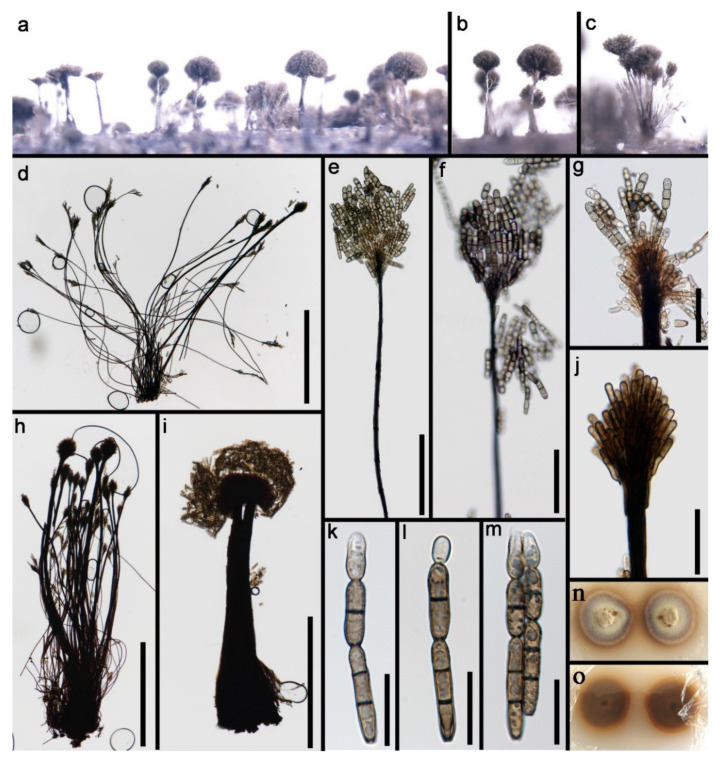
*Vamsapriya aquatica* (HKAS 115791). (**a**–**c**) Colonies on submerged decaying wood; (**d**–**f**,**h**,**i**) Conidiophores with conidia; (**g**) Conidiogenous cells with conidia; (**j**) Conidiogenous cells; (**k**–**m**) conidia;; (**n**,**o**) Culture on PDA from surface and reverse. Scale bars: (**d**,**h**,**i**) = 30 µm; (**e**,**f**) = 100 µm; (**g**,**j**) = 50 µm; (**k**–**m**) = 20 µm.

Xylariales Nannf., Nova Acta R. Soc
Xylariaceae Tul. and C. Tul
*Vamsapriya* Gawas and Bhat, Mycotaxon 94: 150 (2006)
*Vamsapriya aquatica* D.F. Bao, H.Y. Su, K.D. Hyde and Z.L. Luo, sp. nov. (Figure 25).
Index Fungorum number: IF 558598; Faces of fungi number: FoF 09920
Holotype—HKAS 115791
Etymology—Referring the fungus was collected form aquatic habitat.

*Saprobic* on decaying wood submerged in freshwater habitats. **Sexual morph:** Undetermined. **Asexual morph:** *Colonies* effuse on natural substrate and dark brown to black. *Mycelium* immersed, composed of branched, septate and brown hyphae. *Conidiophores* 450–947 μm long, 97–177 μm wide at the base, 60–100 μm wide in the middle, 40–120 μm wide at the apical fertile region, macronematous, synnematous, branched, septate, brown to dark brown and smooth. *Synnemata* erect, rigid, velvety, dark brown, smooth and composed of compact, parallel, adpressed conidiophores. *Conidiogenous cells* 4.5–6.5 × 1.8–2.2 µm, (x = 5.3 × 2.0 μm, *n* = 30), monotretic, enteroblastic, terminal, discrete, ellipsoidal, brown to dark brown and smooth. *Conidia* 16.5–33 × 5–6 µm, (x = 24.5 × 5.5 μm, *n* = 35), catenate, initially subhyaline to pale brown, brown to dark brown when mature, minutely verrucose, cylindrical to obclavate, rounded at the apex, truncate at base, straight or slightly curved, mostly 2-septate, up to 4-septate at maturity and slightly constricted at the septa, smooth. 

Culture characteristics: Conidia germinated on PDA within 24 h and colonies grew on MEA, reaching 25–30 mm in three weeks at 25 °C, dense, floccose, dry, raised from the side view and entire edge, greyish yellow at the middle from above, pale yellow at the margin, dark yellow at the middle from below, pale yellow at the margin and not producing pigmentation in culture. 

Material examined: Nujiang River, Yunnan Province, China on submerged bamboo culms, acquired in May 2016, Z.L. Luo, S-970 (HKAS 115791, holotype), ex-type living culture, DLUCC 970. GenBank accession numbers: (ITS) MZ420740,

Notes: *Vamsapriya aquatica* is morphologically quite similar to *V. indica*. *V. aquatica* shares some similar characters with *V. indica*, such as, macronematous, synnematous, septate, branched, brown to dark brown, conidiophores, monotretic, enteroblastic, terminal *Conidiogenous cells* and catenate, cylindrical and septate conidia. However, *V. aquatica* can be distinguished from *V. indica* by the shorter conidiophores (450–947 vs. 700–1100 μm), smaller conidia (16.5–33 × 5–6 vs. 35–290 × 4–6.5 μm) and less conidial septum (1–3-septate, mostly 2-septate vs. 1–3-septate when young, more than 20-septate at maturity) [117]. In addition, we compared the nucleotides of ITS region between *V. aquatica* and *V. indica*; there were 39 bp (6.5%) differences, which is strongly support our species to be a new species.

**Figure 26:**
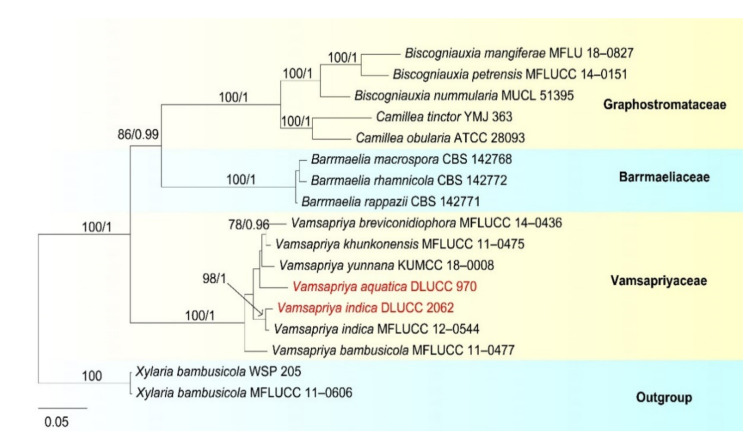
RAxML tree based on analysis of combined LSU, ITS and RPB2 dataset The combined analyses include 17 strains with 2724 characters including gaps (LSU: 886 bp, ITS: 663 bp and RPB2: 1175 bp). The tree is rooted to *Xylaria bambusicola* (WSP 205 and MFLUCC 11–0606). Tree topology of the maximum likelihood analysis and Bayesian analysis are similar. The RAxML analysis of the combined dataset yielded a best scoring tree (Figure 26) with a final ML likelihood value of −11,166.355281. The matrix had 886 distinct alignment patterns, with 27.78% undetermined characters or gaps. Estimated base frequencies were as follows: A = 0.256040, C = 0.243930, G = 0.259986, T = 0.240043; substitution rates AC = 1.257713, AG = 3.660697, AT = 1.322463, CG = 0.963566, CT = 7.100810, GT = 1.000000; gamma distribution shape parameter α = 0.218597. Bootstrap values for maximum likelihood (ML) ≥ 75% and clade credibility values ≥ 0.95 from Bayesian-inference analysis labelled on the nodes. The newly obtained sequence is indicated in red.

Phylogenetic analysis showed that *Vamsapriya aquatica* close to *V. yunnana* (Figure 26). However, *V. aquatica* differs from *V. yunnana* by the conidial shape (cylindrical to obclavate truncate at base, rounded at the apex vs. fusiform, broad at middle, obtuse at base, rostrate at tip) and size (16.5–33 × 5–6 vs. 50–70 × 9–11 μm) [118]. Thus, we introduce the new collection as a new species.

**Figure 27:**
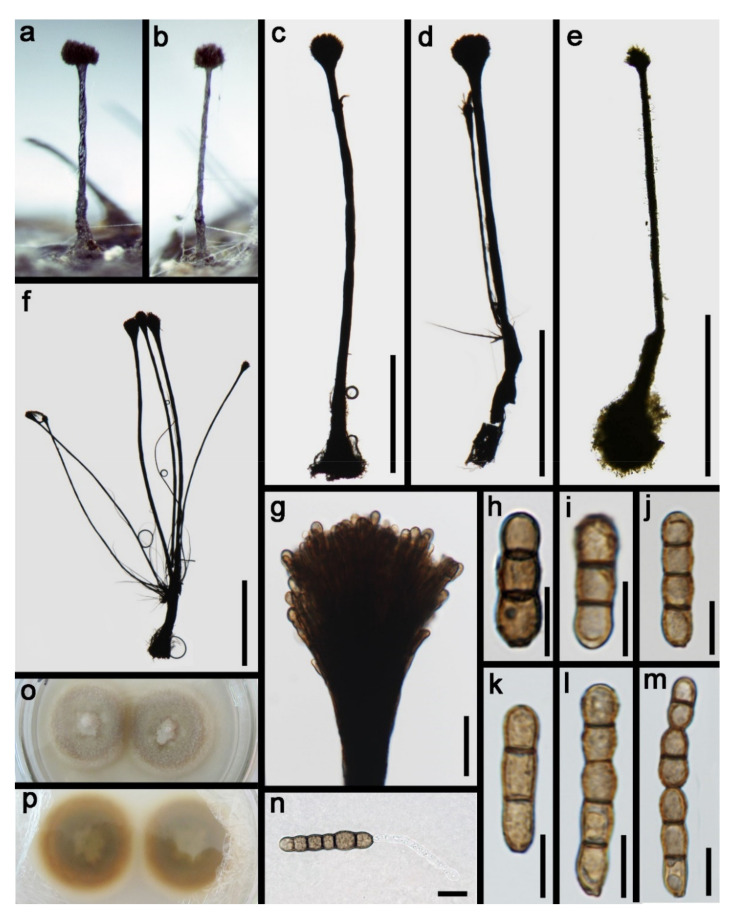
*Vamsapriya indica* (HKAS 115803). (**a**,**b**) Colonies on submerged decaying wood; (**c**–**f**) Conidiophores; (**g**) Conidiogenous cells; (**h**–**m**) Conidia; (**n**) Germinating conidia; (**o**,**p**) Culture on PDA from surface and reverse. Scale bars: (**c**–**f**) 500 µm; (**g**) 20 µm (**h**–**n**) 10 µm.

*Vamsapriya indica* Gawas & Bhat, Mycotaxon 94: 150 (Figure 27).

*Saprobic* on decaying wood submerged in freshwater habitats. Sexual morph: Undetermined. **Asexual morph**: *Colonies* effuse on natural substrate and dark brown to black. *Mycelium* mostly immersed in the substratum, composed of subhyaline, septate, branched and smooth hyphae. *Conidiophores* 1145–1475 μm long, 105–235 μm wide at the base, 50–80 μm wide in the middle, 70–155 μm wide at the apical fertile region, macronematous, synnematous, cylindrical, straight, or slightly flexuous, septate, smooth, dark brown and unbranched. *Synnemata* erect, rigid, dark brown and composed of compact parallel conidiophores. *Conidiogenous cells* 5–9 × 3–5 μm (= 20 × 5 μm, *n* = 30), monotretic, integrated or discrete, terminal, clavate, slightly curved toward the exterior. *Conidia* 15–30 × 4–6.5 μm (= 20 × 5 μm, *n* = 30) acrogenous, catenate, dry, cylindrical to obclavate, straight, 1-4-septate, constricted at the septa, brown dark brown, smooth to slightly verrucose and developing acropetal chains. 

Culture characteristics: Conidia germinated on PDA within 24 h and colonies grew on MEA, reaching 30–35 mm in three weeks at 25 °C, dense, floccose, dry, raised from the side view with entire edge, pale grey at the middle from above, white to greyish white at the margin, dark yellow at the middle from below, pale yellow at the margin and not producing pigmentation in culture.

Material examined: Yunnan Province, China, and were saprobic on bamboo culms submerged in Lancang River, acquired in May 2017, Z.L. Luo, S-2062 (HKAS 115803), living culture, DLUCC 2062. GenBank accession numbers: (LSU) MZ420762, (SSU) MZ420751, (ITS) MZ420747, (tef-1) MZ442694, (RPB2) MZ442699.

Notes: The new collection is identified as *Vamsapriya indica* based on morphology and phylogeny. In the phylogenetic analysis, our new isolate clustered with the ex-type of *V. indica* (MFLUCC 12–0544) with strong bootstrap support (95 ML/1.00 PP, Figure 25). Morphology of our collection is almost identical to the holotype of *V. indica*, except for the conidial size; the conidia of holotype are much longer than our collection (DLUCC 2062).

*Vamsapriya indica* was introduced by Dai et al. [117] and was collected on bamboo culms in Thailand and India. While our collection was collected from freshwater habitats in China, this is the first report of this species from China.

### 3.2. Biodiversity of Freshwater Fungi in China

**Figure 28:**
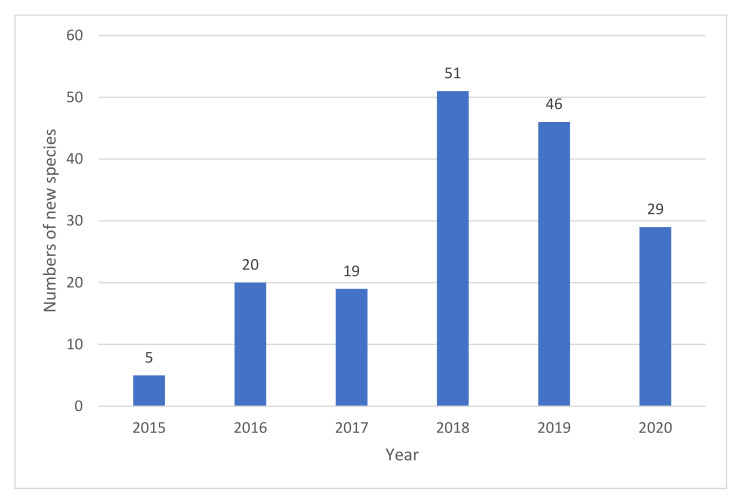
Number of new freshwater fungi reported from China from 2015–2020.

Hu et al. [63] documented the biodiversity of aquatic fungi in China and reported 782 freshwater fungi from 24 provinces/districts of China. In recent years, many new species have been introduced from China and over the last five years, studies of freshwater fungi in China have been rapidly increasing. In this paper, a checklist of freshwater fungi in China from 2015–2020 is provided (Table S1). A total 243 freshwater fungi have been described in China during last five years, of which 170 species are new species (Figure 28). Thus, until 2020, about 1025 freshwater fungi have been reported from China.

Freshwater fungi reported in China are highly diverse in Ascomycota, a few are Zygomycetes [63]. All the 243 freshwater fungi reported in China belong to Ascomycota as follows: 241 species belong to 56 families and 25 orders, of which 25 families and 5 orders belong to Dothideomycetes, one family and one order belong to Eurotiomycetes, 30 families and 19 orders belong to Sordariomycetes, two species have been referred to as ascomycota *incertae sedis* (Table S1). The most common orders in Dothideomycetes are Pleosporales, followed by Tubeufiales, while the common orders in Sordariomycetes are Chaetosphaeriales, followed by Pleurotheciales and Hypocreales (Table S1).

**Figure 29:**
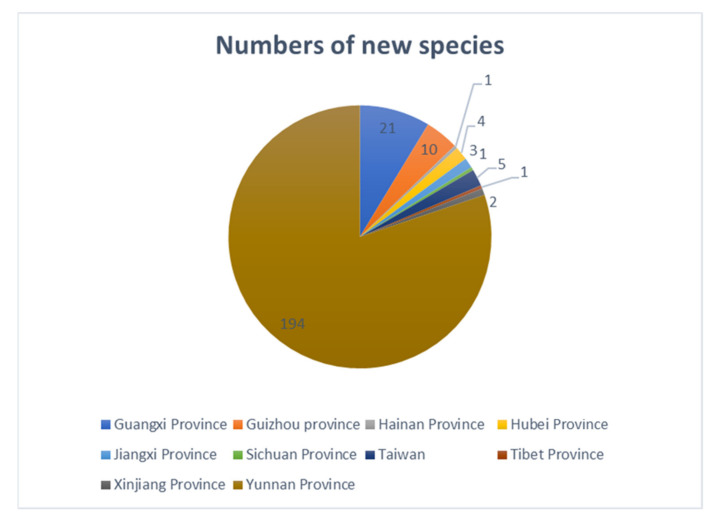
Number of freshwater fungi reported in each province of China during 2015–2020.

During the last five years, studies of freshwater fungi in China have mainly been focused on southern China (Yunnan, Guizhou, Guangxi, Hainan, Jiangxi, Sichuan and Tibet province, Figure 29), while other regions are poorly reported. In southern China, most species were reported from Yunnan province (194 species), flowed by Guangxi (21 species), Guizhou (10 species), Hainan (1species), Jiangxi (3 species), Sichuan (1 species) and Tibet provinces (1 species). Few species were reported from other regions of China, e.g., Hubei, Jiangxi, Xinjiang provinces and Taiwan (Figure 29), and this points out the regional limitations in research on freshwater fungi in China. Freshwater fungi in many regions of China are still to be studied and the current trend suggests that a large proportion of new species awaits discovery in China.

In the present study, we report sixteen freshwater hyphomycetes from Thailand and China. Six new species, six new country records (four new records for China and two new records for Thailand), one new combination and three new collections are introduced. Of the sixteen freshwater hyphomycetes, thirteen of them are Sordariomycetes and three are Dothideomycetes. These taxa are from twelve genera, namely. *Aquapteridospora*, *Acrodictys*, *Chloridium*, *Neospadicoides*, *Pseudoberkleasmium*, *Pseudodactylaria*, *Pleomonodictys*, *Sporidesmium*, *Sporoschisma*, *Tetraploa*, *Vamsapriya* and *Wongia*, of which two genera, *Aquapteridospora* and *Neospadicoides* were exclusively from freshwater habitats and were known only from China and Thailand [28,29]. *Acrodictys*, *Chloridium*, *Pseudoberkleasmium*, *Pseudodactylaria*, *Sporidesmium*, *Sporoschisma* and *Tetraploa* are commonly found freshwater habitats. *Pleomonodictys* and *Vamsapriya* were first reported from freshwater habitats and China in this study [94,95,117,118,119,120,121]. 

## 4. Discussion

Freshwater hyphomycetes are a highly diverse group, with a worldwide distribution. They can adapt to different habitats and environments. They have unique adaptions to adapt and survive in the freshwater habitats with the distinct conidia morphologies [122]. The conidia of freshwater hyphomycetes have diverse shapes and sizes. For example, most Ingoldian fungi have tetraradiate, branched or filiform conidia and these can help attach to surfaces and colonize substrates [6,123,124]. When a tetraradiate spore attaches to the surface of a substrate, it attaches at three points, with the spore acting as a tripod, representing a very stable attachment form [123]. Some species have conidia with prominent sheaths or appendages and these characters strengthen adhesion to surfaces of substrate, which also helps to prevent conidial detachment by strong water currents [124,125]. Freshwater hyphomycetes are dominant fungi on submerged leaves and woody substrates. The main advantage of these fungi on submerged leaves and woody substrates is that they have the ability to maintain activity at low temperatures and degrade submerged organic matter under various climatic conditions [126,127,128].

Traditional identification of freshwater hyphomycetes was mainly based on morphological characters but with the advent of DNA sequence analyses, the identification and classification of freshwater hyphomycetes have greatly improved with better insights into species complexes and linking sexual–asexual morphs as well resolving problems associated with dual nomenclature. Freshwater hyphomycetes are distributed in different phyla, classes and orders. Despite some species having similar morphologies, phylogenetic analyses showed that they are polyphyletic and associated with different orders, families, and classes, e.g., *Acrodictys*-like taxa and *Sporidesmium*-like taxa [101,124]. *Sporidesmium* is a typical example to show the polyphyletic nature of freshwater hyphomycetes, the genus was redescribed by Ellis [97] with a broad generic concept, characterized by solitary or gregarious conidiophores, monoblastic, determinate or percurrent conidiogenous cells, cylindrical, fusiform, obclavate, obpyriform and sometimes rostrate conidia [71,97,99,129]. Later studies have segregated *Sporidesmium* in several different genera, namely *Ellisembia*, *Imimyces*, *Linkosia*, *Penzigomyces*, *Polydesmus*, *Repetophragma*, *Sporidesmiella* and *Stanjehughesia* based on morphological characters [130,131]. Recent studies found that *Sporidesmium* is polyphyletic and related to different orders and families in Dothidieomycetes and Sordariomycetes [71,101,102,132]. Our study herein reports similar results.

In this study, three *Sporidesmium* species are introduced. Phylogenetic analyses showed that the three species grouped in three different clades within Diaporthomycetidae *S. aturbinatum* were within Sporidesmiaceae with strong bootstrap support (Figure 13), while *S. tropicale* and *S. nujiangense* were phylogenetically distant from Sporidesmiaceae in two other clades. *S. tropicale* clustered as a sister taxon to Bullimycetaceae with low support, which is consistent with the analyses of Yang et al. [101]. The placement of this species is still questionable and further phylogenetic analyses are needed to resolve its placement. The novel species, *Sporidesmium nujiangense* clustered with *S. olivaceoconidium*, *S. aquaticivaginatum* and *S. guizhouense* in a stable and well-supported clade sister to Distoseptisporales. Yang et al. [101] showed that *S. olivaceoconidium* and *S. aquaticivaginatum* are basal to Sporidesmiaceae with low support, while, in the analyses of Liu et al. [102], *S*. *olivaceoconidium* and *S. aquaticivaginatum* and *S. guizhouense* formed a stable clade close to Jobellisiales. Morphology of these taxa are not that different from other taxa in the Sporidesmiaceae; however, they are phylogenetically distinct.

Freshwater fungi in Thailand have been studied for several decades [18,47,48,50,53,133]. Calabon et al. [134] summarized the studies of freshwater fungi in Thailand during 2015–2020 with a total 129 new species. Fungal numbers have been estimated between 2.2 and 3.8 million; however, only around 2.6 and 4.5% species of fungi and fungus-like taxa have been accepted [135]. Over the past five years, 298 new species have been reported from China and Thailand, which indicates a high diversity of freshwater fungi, and many new species are still not yet discovered. Recently, the new generation sequencing (NGS) method has been used to better assess fungal diversity, and this greatly improved our understanding on the diversity, ecology and distribution of fungi [136,137,138,139,140]. Studies of freshwater fungi are mainly focused on lignicolous freshwater fungi but fungi on other hosts are poorly reported. The diversity of freshwater fungi is much higher than previously thought. Using NGS method to investigate freshwater fungal diversity may help for a better understanding of their ecology and distribution.

## Data Availability

All sequences generated in this study were submitted to GenBank.

## Supplementary Materials

The following are available online at https://www.mdpi.com/article/10.3390/jof7080669/s1, Table S1: Checklist of freshwater fungi in China from 2015–2020.
