# Supplementary material for: Biodiversity of Lignicolous Freshwater Hyphomycetes from China and Thailand and Description of Sixteen Species
Source: J Fungi (Basel). 2021 Aug 18;7(8):669. doi: 10.3390/jof7080669 (PMC8399276; doi:10.3390/jof7080669)
Supplement: Supplementary file 1 [file jof-07-00669-s001.zip › jof-1339657-supplementary.pdf]

Table S1. Checklist of freshwater fungi in China from 2015–2020.

Species	Province	Year	References
**JAHNULALES**			
**Aliquandostipitaceae**			
Ascagilis guttulaspora	Guangxi	2017	[1]
Clohesyomyces aquaticus	Yunnan	2020	[2]
Jahnula rostrata	Yunnan	2020	[2]
**KIRSCHSTEINIOTHELIALES**			
**Kirschsteiniotheliaceae**			
Kirschsteiniothelia aquatica	Yunnan	2018	[3]
Kirschsteiniothelia atra	Yunnan	2016	[4]
Kirschsteiniothelia cangshanensis	Yunnan	2018	[3]
Kirschsteiniothelia fluminicola	Yunnan	2018	[3]
Kirschsteiniothelia rostrata	Yunnan	2018	[3]
Kirschsteiniothelia submersa	Yunnan	2016	[4]
**MINUTISPHAERALES**			
**Acrogenosporaceae**			
Acrogenospora aquatica	Yunnan	2020	[5]
Acrogenospora basalicellularispora	Yunnan	2020	[5]
Acrogenospora ellipsoidea	Yunnan	2020	[5]
Acrogenospora guttulatispora	Yunnan	2020	[5]
Acrogenospora obovoidspora	Yunnan	2020	[5]
Acrogenospora olivaceospora	Yunnan	2020	[5]
Acrogenospora sphaerocephala	Yunnan	2020	[5]
Acrogenospora submersa	Yunnan	2020	[5]
Acrogenospora subprolata	Yunnan	2020	[5]
Acrogenospora verrucispora	Yunnan	2020	[5]
Acrogenospora yunnanensis	Yunnan	2020	[5]
**PLEOSPORALES**			
**Amniculicolaceae**			
Amniculicola aquatica	Yunnan	2019	[6]
Amniculicola guttulata	Yunnan	2019	[6]
Murispora aquatica	Yunnan	2019	[7]
Murispora cicognanii	Yunnan	2019	[6]
Murispora fagicola	Tibet	2019	[7]
**Bambusicolaceae**			
Bambusicola aquatica	Yunnan	2020	[2]
**Caryosporaceae**			
Caryospora aquatica	Yunnan	2020	[2]
**Corynesporascaceae**			
Corynespora lignicola	Yunnan	2020	[8]
Corynespora submersa	Yunnan	2020	[8]
**Dictyosporiaceae**			
Aquadictyospora lignicola	Yunnan	2017	[9]
Dictyocheirospora aquatica	Yunnan	2016	[10]
Dictyocheirospora garethjonesii	Yunnan	2016	[10]
Dictyocheirospora rotunda	Yunnan	2016	[10]
Dictyocheirospora tetraploides	Yunnan	2018	[11]
Dictyosporium muriformis	Yunnan	2020	[8]

Jalapriya pulchra	Yunnan	2016	[12]
Pseudodictyosporium wauense	Yunnan	2017	[9]
Vikalpa lignicola	Yunnan	2016	[12]
**Lentitheciaceae**			
Lentithecium cangshanense	Yunnan	2016	[13]
Lentithecium kunmingense	Yunnan	2020	[2]
**Lindgomycetaceae**			
Aquimassariosphaeria kunmingensis	Yunnan	2020	[2]
**Lophiostomataceae**			
Biappendiculispora japonica	Yunnan	2019	[14]
Flabellascoma aquaticum	Yunnan	2019	[14]
Flabellascoma fusiforme	Yunnan	2019	[14]
Neovaginatispora fuckelii	Yunnan	2019	[14]
Pseudocapulatispora longiappendiculata	Yunnan	2020	[2]
Sigarispora clavata	Yunnan	2019	[14]
**Massarinaceae**			
Helminthosporium aquaticum	Yunnan	2016	[15]
Helminthosporium submersum	Yunnan	2018	[16]
Helminthosporium velutinum	Yunnan	2016	[15]
**Melanommataceae**			
Camposporium appendiculatum	Yunnan	2020	[8]
Camposporium appendiculatum	Yunnan	2020	[8]
Camposporium multiseptatum	Yunnan	2020	[8]
Camposporium multiseptatum	Yunnan	2020	[8]
Camposporium pellucidum	Yunnan	2020	[8]
Phragmocephala atra	Yunnan	2015	[17]
Phragmocephala garethjonesii	Yunnan	2015	[17]
**Morosphaeriaceae**			
Aquihelicascus yunnanensis	Yunnan	2020	[2]
Helicascus alatus	Yunnan	2018	[18]
Neohelicascus aquaticus	Yunnan	2018	[2]
Neohelicascus submersus	Yunnan	2020	[2]
**Neophaeosphaeriaceae**			
Neophaeosphaeria phragmiticola	Yunnan	2018	[19]
**Nigrogranaceae**			
Nigrograna cangshanensis	Yunnan	2017	[20]
**Occultibambusaceae**			
Occultibambusa kunmingensis	Yunnan	2020	[2]
Occultibambusa pustula	Yunnan	2020	[2]
Seriascoma didymosporum	Yunnan	2020	[2]
**Periconiaceae**			
Periconia aquatica	Yunnan	2017	[21]
Periconia cookei	Yunnan	2018	[19]
Periconia minutissima	Yunnan	2017	[21]
Periconia pseudobyssoides	Yunnan	2018	[19]
Periconia submersa	Yunnan	2017	[21]
**Phaeoseptaceae**			
Pleopunctum ellipsoideum	Guizhou	2019	[68]
Pleopunctum pseudoellipsoideum	Guizhou	2019	[68]
**Pleosporaceae**			
Curvularia eragrostidis	Yunnan	2015	[17]

Curvularia verruculosa	Yunnan	2015	[17]
**Roussoellaceae**			
Neoroussoella bambusae	Yunnan	2020	[2]
Neoroussoella leucaenae	Yunnan	2020	[2]
Roussoella aquatica	Yunnan	2020	[2]
**Tetraplosphaeriaceae**			
Tetraploa aquatica	Guizhou	2020	[23]
Tetraploa puzheheiensis	Yunnan	2020	[2]
Tetraploa yunnanensis	Yunnan	2020	[2]
**Torulaceae**			
Dendryphion aquaticum	Yunnan	2016	[4]
Dendryphion fluminicola	Yunnan	2018	[24]
Dendryphion nanum	Yunnan	2016	[4]
Dendryphion submersum	Yunnan	2016	[4]
Neotorula aquatica	Yunnan	2016	[4]
Neotorula submersa	Yunnan	2016	[25]
Rostriconidium aquaticum	Yunnan	2018	[24]
Torula aquatica	Yunnan	2018	[24]
Torula fici	Yunnan	2018	[24]
Torula ficus	Yunnan	2018	[4]
Torula gaodangensis	Guizhou	2017	[21]
Torula masonii	Yunnan	2018	[24]
**TUBEUFIALES**			
**Tubeufiaceae**			
Berkleasmium aquaticum	Guangxi	2017	[26]
Berkleasmium guangxiense	Guangxi	2017	[26]
Dematiohelicoma perelegans	Hainan	2018	[27]
Helicoma multiseptatum	Guangxi	2018	[27]
Helicomyces chiayiensis	Taiwan	2018	[28]
Helicomyces hyalosporus	Guangxi	2018	[27]
Helicomyces roseus	Yunnan	2017	[29]
Muripulchra aquatica	Yunnan	2017	[29]
Neohelicomyces aquaticus	Yunnan	2017	[29]
Neohelicomyces dehongensis	Yunnan	2020	[2]
Neohelicomyces grandisporus	Yunnan	2017	[29]
Neohelicomyces hyalosporus	Guangxi	2018	[27]
Neohelicomyces submersus	Yunnan	2017	[29]
Neohelicosporium aquaticum	Guangxi	2018	[30]
Neohelicosporium guangxiense	Guangxi	2017	[30]
Neohelicosporium hyalosporum	Guangxi	2018	[30]
Neohelicosporium parvisporum	Guangxi	2018	[30]
Neohelicosporium taiwanense	Taiwan	2018	[28]
Pseudohelicomyces hyalosporus	Yunnan	2018	[27]
Pseudohelicoon subglobosum	Taiwan	2018	[31]
Tubeufia aquatica	Yunnan	2017	[29]
Tubeufia cylindrothecia	Yunnan	2017	[29]
Tubeufia eccentrica	Guangxi	2018	[27]
Tubeufia fangchengensi	Guangxi	2018	[27]
Tubeufia geniculata	Taiwan	2018	[32]
Tubeufia guangxiensis	Guangxi	2017	[33]
Tubeufia hechiensis	Guangxi	2018	[27]

Tubeufia inaequalis	Guangxi	2018	[27]
Tubeufia machaerinae	Guangxi	2018	[27]
Tubeufia rubra	Guangxi	2018	[27]
Tubeufia sympodihylospora	Guangxi	2018	[27]
Tubeufia taiwanensis	Taiwan	2018	[27]
Tubeufia xylophila	Guangxi	2018	[27]
**EUROTIOMYCETES**			
**CHAETOTHYRIALES**			
**Herpotrichiellaceae**			
Minimelanolocus aquaticus	Yunnan	2015	[34]
Minimelanolocus asiaticus	Yunnan	2015	[34]
Minimelanolocus curvatus	Yunnan	2015	[34]
Minimelanolocus melanicus	Yunnan	2015	[34]
Minimelanolocus obscuru	Yunnan	2015	[34]
Minimelanolocus yunnanensis	Yunnan	2016	[35]
**SORDARIOMYCETES**			
**AMPHISPHAERIALES**			
**Apiosporaceae**			
Arthrinium aquaticum	Yunnan	2019	[36]
**Sporocadaceae**			
Seiridium aquaticum	Yunnan	2019	[36]
**ATRACTOSPORALES**			
**Atractosporaceae**			
Atractospora aquatica	Yunnan	2019	[36]
**BARBATOSPHAERIALES**			
**Barbatosphaeriaceae**			
Barbatosphaeria lignicola	Yunnan	2019	[36]
**CHAETOSPHAERIALES**			
**Chaetosphaeriaceae**			
Brunneodinemasporium jonesii	Guangxi	2016	[37]
Chaetosphaeria aquatica	Yunnan	2019	[36]
Chaetosphaeria catenulata	Yunnan	2019	[36]
Chaetosphaeria cubensis	Yunnan	2019	[36]
Chaetosphaeria guttulata	Yunnan	2019	[36]
Chaetosphaeria myriocarpa	Yunnan	2019	[36]
Chaetosphaeria submersa	Yunnan	2019	[36]
Chloridium gonytrichii	Yunnan	2019	[36]
Codinaea yunnanensis	Yunnan	2019	[36]
Dictyochaeta cangshanensis	Yunnan	2019	[36]
Dictyochaeta ellipsoidea	Yunnan	2019	[36]
Dictyochaeta lignicola	Yunnan	2019	[36]
Dictyochaeta submersa	Yunnan	2019	[36]
Nawawia oviformis	Sichuan	2016	[37]
Sporoschisma aquaticum	Yunnan	2016	[39]
Sporoschisma hemipsila	Yunnan	2016	[39]
Sporoschisma juvenile	Yunnan	2019	[36]
Sporoschisma mirabile	Yunnan	2016	[39]
Sporoschisma nigroseptatum	Yunnan	2016	[39]
Sporoschisma phaeocentri	Yunnan	2016	[39]
Sporoschisma taitense	Yunnan	2016	[39]
Tainosphaeria jonesii	Guangxi	2016	[37]

Tainosphaeria lunata	Yunnan	2019	[36]
**CONIOCHAETALES**			
**Cordanaceae**			
Cordana aquatica	Yunnan	2019	[36]
Cordana lignicola	Yunnan	2019	[36]
Cordana terrestris	Yunnan	2019	[36]
**CONIOSCYPHALES**			
**Conioscyphaceae**			
Conioscypha verrucosa	Guizhou	2020	[8]
Conioscypha aquatica	Yunnan	2019	[36]
Conioscypha submersa	Yunnan	2019	[36]
**DIAPORTHOMYCETIDAE families *incertae sedis***			
**Acrodictyaceae**			
Acrodictys fluminicola			
**Junewangiaceae**			
Dictyosporella hydei	Yunnan	2018	[40]
Junewangia aquatica	Yunnan	2018	[41]
Sporidesmiella aquatica	Yunnan	2019	[36]
Sporidesmiella hyalosperma	Yunnan	2019	[36]
Sporidesmiella novae-zelandiae	Yunnan	2019	[36]
Wongia aquatica	Yunnan	2019	[36]
**Pseudostanjehughesiaceae**			
Pseudostanjehughesia lignicola	Yunnan	2019	[36]
**Rhamphoriaceae**			
Rhodoveronaea aquatica	Yunnan	2019	[36]
**DISTOSEPTISPORALES**			
**Aquapteridosporaceae**			
Aquapteridospora fusiformis	Yunnan	2019	[36]
**Distoseptisporaceae**			
Distoseptispora aquatica	Yunnan	2016	[4]
Distoseptispora cangshanensis	Yunnan	2018	[42]
Distoseptispora fluminicola	Yunnan	2016	[4]
Distoseptispora obpyriformis	Yunnan	2018	[42]
Distoseptispora rostrata	Yunnan	2018	[42]
Distoseptispora submersa	Yunnan	2018	[42]
Distoseptispora suoluoensis	Guizhou	2018	[43]
**GLOMERELLALES**			
**Reticulascaceae**			
Cylindrotrichum clavatum	Yunnan	2018	[44]
Cylindrotrichum gorii	Yunnan	2018	[44]
Cylindrotrichum submersum	Yunnan	2019	[36]
Kylindria aquatica	Yunnan	2018	[44]
Kylindria chinensis	Yunnan	2018	[44]
**HYPOCREALES**			
**Nectriaceae**			
Aquanectria jacinthicolor	Yunnan	2018	[45]
Aquanectria penicillioides	Yunnan	2019	[36]
Chaetopsina beijingensis	Yunnan	2019	[36]
Cosmospora aquatica	Yunnan	2019	[36]
Cosmosporella olivacea	Xinjiang	2018	[45]
Fusicolla aquaeductuum	Xinjiang	2018	[45]

Mariannaea chlamydospora	Hubei	2017	[46]
Mariannaea cinerea	Yunnan	2017	[46]
Mariannaea fusiformis	Hubei	2017	[46]
Mariannaea fusiformis	Hubei	2017	[46]
Mariannaea lignicola	Jiangxi	2017	[46]
Mariannaea samuelsii	Yunnan	2019	[36]
Mariannaea superimposita	Yunnan	2019	[36]
Paracremonium binnewijzendii	Yunnan	2019	[36]
Stachybotrys chartarum	Yunnan	2019	[36]
Stachybotrys chlorohalonata	Yunnan	2019	[36]
**SORDARIOMYCETES *incertae sedis***			
Pseudoconlarium punctiforme	Guizhou	2020	[8]
**Ceratosphaeriaceae**			
Ceratosphaeria aquatica	Yunnan	2019	[36]
**Magnaporthaceae**			
Aquafiliformis lignicola	Yunnan	2019	[36]
**MYRMECRIDIALES**			
**Myrmecridiaceae**			
Myrmecridium aquaticum	Yunnan	2019	[36]
Neomyrmecridium guizhouense	Guizhou	2020	[8]
**PLEUROTHECIALES**			
**Pleurotheciaceae**			
Pleurothecium pulneyense	Yunnan	2018	[47]
Pleurothecium recurvatum	Yunnan	2018	[47]
Phaeoisaria clematidis	Yunnan	2018	[47]
Phaeoisaria guttulata	Guizhou	2018	[47]
Pleurotheciella uniseptata	Yunnan	2018	[47]
Sterigmatobotrys uniseptata	Yunnan	2019	[36]
Phaeoisaria aquatica	Yunnan	2018	[47]
Pleurotheciella aquatica	Yunnan	2018	[47]
Pleurotheciella fusiformis	Yunnan	2018	[47]
Pleurotheciella guttulata	Yunnan	2018	[47]
Pleurotheciella lunata	Yunnan	2018	[47]
Pleurotheciella saprophytica	Yunnan	2018	[47]
Pleurotheciella submersa	Yunnan	2018	[47]
Pleurothecium aquaticum	Yunnan	2018	[47]
**PSEUDODACTYLARIALES**			
**Pseudodactylariaceae**			
Pseudodactylaria fusiformis	Guizhou	2020	[48]
**Savoryellales**			
**Savoryellaceae**			
Dematiosporium aquaticum	Yunnan	2018	[36]
**SORDARIALES**			
**Chaetomiaceae**			
Chaetomium globosum	Yunnan	2019	[36]
**Lasiosphaeriaceae**			
Apiosordaria hamata	Hubei	2016	[49]
Cercophora caudata	Yunnan	2019	[36]
**SORDARIALES genera *incertae sedis***			
Cuspidatispora xiphiago	Yunnan	2019	[36]
**SPORIDESMIALES**			

Sporidesmium cangshanense	Yunnan	2016	[4]
Sporidesmium fluminicola	Yunnan	2016	[4]
Sporidesmium submersum	Yunnan	2016	[4]
Sporidesmium dulongense	Yunnan	2020	[6]
Sporidesmium guizhouense	Yunnan	2018	[50]
Sporidesmium lageniforme	Yunnan	2019	[36]
Sporidesmium lignicola	Yunnan	2019	[36]
**TOGNINIALES**			
**Togniniaceae**			
Phaeoacremonium ovale	Yunnan	2018	[51]
**XENOSPADICOIDALES**			
**Xenospadicoidaceae**			
Neospadicoides aquatica	Yunnan	2019	[36]
Neospadicoides lignicola	Yunnan	2019	[36]
Neospadicoides yunnanensis	Yunnan	2019	[36]
Neospadicoides yunnanensis	Yunnan	2019	[36]
**XYLARIALES**			
**Hypoxylaceae**			
Hypoxylon lignicola	Yunnan	2019	[36]
**ASCOMYCOTA *incertae sedis***			
Phaeomonilia aquatica	Jiangxi	2018	[52]
Xylohyphopsis aquatica	Jiangxi	2016	[53]

References

1. Hyde, K.D.; Norphanphoun, C.; Abreu, V.P.; Bazzicalupo, A.; Chethana, K.W.T.; Clericuzio, M.; Dayarathne, M.C.; Dissanayake, A.J.; Ekanayaka, A.H.; He, M.Q.; et al. Fungal diversity notes 603–708: Taxonomic and phylogenetic notes on genera and species. *Fungal Divers.* **2017**, *87*, 1–235, doi:10.1007/s13225-017-0391-3.
2. Dong, W.; Wang, B.; Hyde, K.D.; McKenzie, E.H.C.; Bhat, D.J.; Raja, H.A.; Tanaka, K.; Abdel-Wahab, M.A.; Abdel-Aziz, F.A.; Doilom, M.; et al. Freshwater Dothideomycetes. *Fungal Divers.* **2020**, *105*, 319–575, doi:10.1007/s13225-020-00463-5.
3. Bao, D.F.; Luo, Z.L.; Liu, J.K.; Bhat, D.J.; Sarunya, N.; Li, W.L.; Su, H.Y.; Hyde, K.D. Lignicolous freshwater fungi in China III: New species and record of *Kirschsteiniothelia* from northwestern Yunnan Province. *Mycosphere* **2018**, *9*, 755–768, doi:10.5943/mycosphere/9/4/4.
4. Su, H.Y.; Hyde, K.D.; Maharachchikumbura, S.S.N.; Ariyawansa, H.A.; Luo, Z.L.; Promputtha, I.; Tian, Q.; Lin, C.G.; Shang, Q.J.; Zhao, Y.C.; et al. The families Distoseptisporaceae fam. nov., Kirschsteiniotheliaceae, Sporormiaceae and Torulaceae, with new species from freshwater in Yunnan Province, China. *Fungal Divers.* **2016**, *80*, 375–409, doi:10.1007/s13225-016-0362-0.
5. Bao, D.F.; McKenzie, E.H.C.; Bhat, D.J.; Hyde, K.D.; Luo, Z.L.; Shen, H.W.; Su, H.Y. *Acrogenospora* (Acrogenosporaceae, Minutisphaerales) appears to be a very diverse genus. *Front. Microbiol*. **2020**, *11*, doi:10.3389/fmicb.2020.01606.
6. Hyde, K.D.; Tennakoon, D.S.; Jeewon, R.; Bhat, D.J.; Maharachchikumbura, S.S.N.; Rossi, W.; Leonardi, M.; Lee, H.M.; Mun, H.Y.; Houbraken, J.; et al. Fungal diversity notes 1036–1150: Taxonomic and phylogenetic contributions on genera and species of fungal taxa. *Fungal Divers.* **2019**, *96*, 1–242, doi:10.1007/s13225-019-00429-2.
7. Bao, D.F.; Wanasinghe, D.N.; Luo, Z.L.; Mortimer, P.E.; Kumar, V.; Su, H.Y.; Hyde, K.D. *Murispora aquatica* sp. nov. and *Murispora fagicola*, a new record from freshwater habitat in China. *Phytotaxa* **2019**, *416*, 1–13, doi:10.11646/phytotaxa.416.1.1.
8. Hyde, K.D.; Dong, Y.; Phookamsak, R.; Jeewon, R.; Bhat, D.J.; Jones, E.B.G.; Liu, N.G.; Abeywickrama, P.D.; Mapook, A.; Wei, D.P.; et al. Fungal diversity notes 1151–1276: Taxonomic and phylogenetic contributions on genera and species of fungal taxa. *Fungal Divers.* **2020**,*100*, 5–277, doi:10.1007/s13225-020-00439-5.
9. Li, W.L.; Luo, Z.L.; Liu, J.K.; Bhat, D.J.; Bao, D.F.; Su, H.Y.; Hyde, K.D. Lignicolous freshwater fungi from China I: *Aquadictyospora lignicola* gen. et sp. nov. and new record of *Pseudodictyosporium wauense* from northwestern Yunnan Province. *Mycosphere* **2017**, *8*, 1587–1597, doi:10.5943/mycosphere/8/10/1.

10. Wang, R.X.; Luo, Z.L.; Hyde, K.D.; Bhat, D.J.; Su, X.J.; Su, H.Y. New species and records of *Dictyocheirospora* from submerged wood in north-western Yunnan, China. *Mycosphere* **2016**, *7*, 1357–1367, doi:10.5943/mycosphere/7/9/9.
11. Yang, J.; Liu, J.K.; Hyde, K.D.; Jones, E.B.G.; Liu, Z.Y. New species in *Dictyosporium*, new combinations in *Dictyocheirospora* and an updated backbone tree for Dictyosporiaceae. *Mycokeys* **2018**, *36*, 83–105, doi:10.3897/mycokeys.36.27051.
12. Boonmee, S.; D'souza, M.J.; Luo, Z.L.; Pinruan, U.; Tanaka, K.; Su, H.Y.; Bhat, D.J.; McKenzie, E.H.C.; Jones, E.B.G.; Taylor, J.E.; et al. Dictyosporiaceae fam. nov. *Fungal Divers*. **2016**, *80*, 457–482, doi:10.1007/s13225-016-0363-z.
13. Su, H.Y.; Luo, Z.L.; Liu, X.Y.; Su, X.J.; Hu, D.M.; Zhou, D.Q.; Bahkali, A.H.; Hyde, K.D. *Lentithecium cangshanense* sp. nov. (Lentitheciaceae) from freshwater habitats in Yunnan Province, China. *Phytotaxa* **2016**, *267*, 61–69, doi:10.11646/phytotaxa.267.1.6.
14. Bao, D.F.; Su, H.Y.; Maharachchikumbura, S.S.N.; Liu, J.K.; Nalumpang, S.; Luo, Z.L.; Hyde, K.D. Lignicolous freshwater fungi from China and Thailand: Multi-gene phylogeny reveals new species and new records in Lophiostomataceae. *Mycosphere* **2019**, *10*, 1080–1099, doi:10.5943/mycosphere/10/1/20.
15. Zhu, D.; Luo, Z.L.; Baht, D.J.; McKenzie, E.H.C.; Bahkali, A.H.; Zhou, D.Q.; Su, H.Y.; Hyde, K.D. *Helminthosporium velutinum* and *H. aquaticum* sp. nov. from aquatic habitats in Yunnan Province. China. *Phytotaxa* **2016**, *253*, 179–190, doi:106.iss4.2015.469.
16. Zhao, N.; Luo, Z.L.; Bhat, D.J.; Liu, J.K.; Bao, D.F.; Hao, Y.E.; Su, H.Y.; Hyde, K.D. *Helminthosporium submersum* sp. nov. (Massarinaceae) from submerged wood in north-western Yunnan Province, China. *Phytotaxa* **2018**, *348*, 269–278, doi:10.11646/phytotaxa.348.4.3.
17. Su, H.Y.; Udayanga, D.; Luo, Z.L.; Manamgoda, D.S.; Zhao, Y.C.; Yang, J.; Liu, X.Y.; Mckenzie, E.H.C.; Zhou, D.Q.; Hyde, K.D. Hyphomycetes from aquatic habitats in Southern China: Species of *Curvularia* (Pleosporaceae) and *Phragmocephala* (Melannomataceae). *Phytotaxa* **2015**, *226*, 201–4216, doi:10.11646/phytotaxa.226.3.1.
18. Zeng, M.; Huang, S.K.; Hyde, K.D.; Zhao, Q. *Helicascus alatus* (Morosphaeriaceae), a new freshwater species from southwestern China. *Phytotaxa* **2018**, *351*, 210–218, doi:10.11646/phytotaxa.351.3.2.
19. Hyde, K.D.; Chaiwan, N.; Norphanphoun, C.; Boonmee, S.; Camporesi, E.; Chethana, K.W.T.; Dayarathne, M.C.; de Silva, N.I.; Dissanayake, A.J.; Ekanayaka, A.H.; et al. Mycosphere notes 169–224. *Mycosphere* **2018**, *9*, 271–430, doi:10.5943/mycosphere/9/2/8.
20. Tibpromma, S.; Hyde, K.D.; Jeewon, R.; Maharachchikumbura, S.S.N.; Liu, J.K.; Bhat, D.J.; Phillips, A.J.L.; Wanasinghe, D.N.; Samarakoon, M.C.; Jayawardena, R.S.; et al. Fungal diversity notes 491–602: Taxonomic and phylogenetic contributions to fungal taxa. *Fungal Divers*. **2017**, *83*, 1–261, doi:10.1007/s13225-017-0378-0.
21. Hyde, K.D.; Norphanphoun, C.; Abreu, V.P.; Bazzicalupo, A.; Chethana, K.W.T.; Clericuzio, M.; Dayarathne, M.C.; Dissanayake, A.J.; Ekanayaka, A.H.; He, M.Q.; et al. Fungal diversity notes 603–708: Taxonomic and phylogenetic notes on genera and species. *Fungal Divers.* **2017**, *87*, 1–235, doi:10.1007/s13225-017-0391-3.
22. Liu, N.G.; Hyde, K.D.; Bhat, D.J.; Jumpathong, J.; Liu, J.K. Morphological and phylogenetic studies of *Pleopunctum* gen. nov. (Phaeoseptaceae, Pleosporales) from China. *Mycosphere* **2019**, *10*, 757–775, doi:10.5943/mycosphere/10/1/17.
23. Li, W.L.; Bao, D.F.; Bhat, D.J.; Su, H.Y. Tetraploa aquatica (Tetraplosphaeriaceae), a new freshwater fungal species from Yunnan Province, China. *Phytotaxa* **2020**, *459*, 181–189, doi:10.11646/phytotaxa.459.2.8.
24. Su, X.J.; Luo, Z.L.; Jeewon, R.; Bhat, D.J.; Bao, D.F.; Li, W.L.; Hao, Y.E.; Su, H.Y.; Hyde, K.D. Morphology and multigene phylogeny reveal new genus and species of Torulaceae from freshwater habitats in northwestern Yunnan, China. *Mycol. Prog.* **2018**, *17*, 531–545.
25. Hyde, K.D.; Hongsanan, S.; Jeewon, R.; Bhat, D.J.; McKenzie, E.H.C.; Jones, E.B.G.; Phookamsak, R.; Ariyawansa, H.A.; Boonmee, S.; Zhao, Q.; et al. Fungal diversity notes 367–490: Taxonomic and phylogenetic contributions to fungal taxa. *Fungal Divers.* **2016**, *80*, 1–270, doi:10.1007/s13225-016-0373-x.
26. Lu, Y.Z.; Boonmee, S.; Liu, J.K.; Hyde, K.D.; Bhat, D.J.; Eungwanichayapant, P.D.; Kang, J.C. Novel *Neoacanthostigma* species from aquatic habitats. *Cryptogam. Mycol.* **2017**, *38*, 169–190, doi:10.7872/crym/v38.iss2.2017.169.
27. Lu, Y.Z.; Liu, J.K.; Hyde, K.D.; Jeewon, R.; Kang, J.C.; Fan, C.; Boonmee, S.; Bhat, D.J.; Luo, Z.L.; Lin, C.G.; et al. A taxonomic reassessment of Tubeufiales based on multi-locus phylogeny and morphology. *Fungal Divers.* **2018**, *92*, 131–344, doi:10.1007/s13225-018-0411-y.
28. Kuo, C.H.; Goh, T.K. Two new species of helicosporous hyphomycetes from Taiwan. *Mycol. Prog.* **2018**, *17*, 557–569, doi:10.1007/s11557-018-1384-7.

29. Luo, Z.L.; Bhat, D.J.; Jeewon, R.; Boonmee, S.; Bao, D.F.; Zhao, Y.C.; Chai, H.M.; Su, H.Y.; Su, X.J.; Hyde, K.D. Molecular phylogeny and morphological characterization of asexual fungi (Tubeufiaceae) from freshwater habitats in Yunnan, China. *Cryptogam. Mycol.* **2017**, *38*, 27–53, doi:10.7872/crym/v38.iss1.2017.27.
30. Lu, Y.Z.; Boonmee, S.; Liu, J.K.; Hyde, K.D.; McKenzie, E.H.C.; Eungwanichayapant, P.D.; Kang, J.C. Multi-gene phylogenetic analyses reveals *Neohelicosporium* gen. nov. and five new species of helicosporous hyphomycetes from aquatic habitats. *Mycol. Prog.* **2018**, *17*, 631–646, doi:10.1007/s11557-017-1366-1.
31. Goh, T.K.; Kuo, C.H. A new species of *Helicoön* from Taiwan. *Phytotaxa* **2018**, *346*, 141–156, doi:10.11646/phytotaxa.346.2.2.
32. Kuo, C.H.; Goh, T.K. A new species and a new record of *Helicomyces* from Taiwan. *Mycoscience* **2018**, *59*, 433–440, doi:10. 1016/j.myc.2018.04.002.
33. Chaiwan, N.; Lu, Y.Z.; Tibpromma, S.; Bhat, D.J.; Hyde, K.D.; Boonmee, S. *Neotubeufia* gen. nov. and *Tubeufia guangxiensis* sp. nov. (Tubeufiaceae) from freshwater habitats. *Mycosphere* **2017**, *8*, 1443–1456, doi:10.5943/mycosphere/8/9/9.
34. Liu, X.Y.; Udayanga, D.; Luo, Z.L.; Chen, L.J.; Zhou, D.Q.; Su, H.Y.; Hyde, K.D. Backbone tree for Chaetothyriales with four new species of *Minimelanolocus* from aquatic habitats. *Fungal Biol.* **2015**, *119*, 1046–1062, doi:10.1016/j.funbio.2015.08.005.
35. Tian, Q.; Doilom, M.; Luo, Z.L.; Chomnunti, P.; Bhat, D.J.; Xu, J.C.; Hyde, K.D. Introducing *Melanoctona tectonae* gen. et sp. nov. and *Minimelanolocus yunnanensis* sp. nov. (Herpotrichiellaceae, Chaetothyriales). *Cryptogam. Mycol.* **2016**, *37*, 477–492, doi:10.11646/phytotaxa.226.3.1.
36. Luo, Z.L.; Hyde, K.D.; Liu, J.K.; Maharachchikumbura, S.S.N.; Jeewon, R.; Bao, D.F.; Bhat, D.J.; Lin, C.G.; Li, W.L.; Yang, J.; et al. Freshwater Sordariomycetes. *Fungal Divers.* **2019**, *99*, 451–660, doi:10.1007/s13225-019-00438-1.
37. Lu, Y.Z.; Liu, J.K.; Hyde, K.D.; Bhat, D.J.; Xiao, Y.P.; Tian, Q.; Wen, T.C.; Boonmee, S.; Kang, J.C. *Brunneodinemasporium jonesii* and *Tainosphaeria jonesii* spp. nov. (Chaetosphaeriaceae, Chaetosphaeriales) from southern China. *Mycosphere* **2016**, *7*, 1323–1332, doi:10.5943/mycosphere/7/9/6.
38. Peng, J.; Chang, D.; Huang, Y.; Yu, Z.F. *Nawawia oviformis* sp. nov. from China. *Mycotaxon* **2016**, *131*, 735–738, doi:10.5248/131.735.
39. Luo, Z.L.; Bao, D.F.; Bhat, D.J.; Yang, J.; Chai, H.M.; Li, S.H.; Bahkali, A.H.; Su, H.Y.; Hyde, K.D. *Sporoschisma* from submerged wood in Yunnan, China. *Mycol. Prog.* **2016**, *15*, 1145–1155, doi:10.1007/s11557-016-1236-2.
40. Song, H.Y.; Huo, G.H.; Hu, D.M. *Dictyosporella hydei* sp. nov., an asexual species from freshwater habitats in China. *Phytotaxa* **2018**, *358*, 181–188, doi:10.11646/phytotaxa.358.2.5.
41. Song, H.Y.; Zhong, P.A.; Liao, J.L.; Wang, Z.H.; Hu, D.M.; Huang, Y.J. *Junewangia aquatica* (Junewangiaceae), a new species from freshwater habitats in China. *Phytotaxa* **2018**, *336*, 272–278, doi:10.11646/phytotaxa.336.3.5.
42. Luo, Z.L.; Hyde, K.D.; Liu, J.K.; Bhat, D.J.; Bao, D.F.; Li, W.L.; Su, H.Y. Lignicolous freshwater fungi from China II: Novel *Distoseptispora* (Distoseptisporaceae) species from northwestern Yunnan Province and a suggested unified method for studying lignicolous freshwater fungi. *Mycosphere* **2018**, *9*, 444–461, doi:10.5943/mycosphere/9/3/2.
43. Yang, J.; Maharachchikumbura, S.S.N.; Liu, J.K.; Hyde, K.D.; Jones, E.B.G.; Al-Sadi, A.M.; Liu, Z.Y. *Pseudostanjehughesia aquitropica* gen. et sp. nov. and *Sporidesmium sensu lato* species from freshwater habitats. *Mycol. Prog.* **2018**, *17*, 591–616, doi:10.1007/s11557-017-1339-4.
44. Maharachchikumbura, S.S.N.; Luo, Z.L.; Su, H.Y.; Al-Sadi, A.M.; Cheewangkoon, R. Reticulascaceae hyphomycetes from submerged wood in Yunnan, China. *Phytotaxa* **2018**, *348*, 187–198, doi:10.11646/phytotaxa.348.3.2.
45. Huang, S.K.; Jeewon, R.; Hyde, K.D.; Bhat, D.J.; Wen, T.C. Novel taxa within Nectriaceae: *Cosmosporella* gen. nov. and *Aquanectria* sp. nov. from freshwater habitats in China. *Cryptog. Mycol.* **2018**, *39*, 169–192, doi:10.7872/crym/v39.iss2.2018.169.
46. Hu, D.M.; Wang, M.; Cai, L. Phylogenetic assessment and taxonomic revision of *Mariannaea*. *Mycol. Prog.* **2017**, *16*, 271–283, doi:10.1007/s11557-016-1252-2.
47. Luo, Z.L.; Hyde, K.D.; Bhat, D.J.; Jeewon, R.; Maharachchikumbura, S.S.N.; Bao, D.F.; Li, W.L.; Su, X.J.; Yang, X.Y.; Su, H.Y. Morphological and molecular taxonomy of novel species Pleurotheciaceae from freshwater habitats in Yunnan, China. *Mycol. Prog.* **2018**, *17*, 511–530, doi:10.1007/s11557-018-1377-6.
48. Lu, Y.Z.; Zhang, J.Y.; Lin, C.G.; Luo, Z.L.; Liu, J.K. *Pseudodactylaria fusiformis* sp. nov. from freshwater habitat in China. *Phytotaxa* **2020**, *446*, 95–102, doi:10.11646/phytotaxa.446.2.2.

49. Wu, B.; Tian, J.Q.; Wang, L.; Liu, J.K.; Hyde, K.D.; Sun, J.Z. *Apiosordaria hamata* sp. nov. from lake sediment in China. *Mycotaxon* **2016**, *131*, 847–857, doi:10.5248/131.847.
50. Liu, L.L.; Yang, J.; Liu, N.G.; Chen, Y.Y.; Gui, X.X.; Liu, Z.Y. *Sporidesmium guizhouense* sp. nov. (Sordariomycetes incertae sedis), a new species from a freshwater habitat in Guizhou Province, China. *Phytotaxa* **2019**, *422*, 144–156, doi:10.11646/phytotaxa.422.2.2.
51. Huang, S.K.; Jeewon, R.; Hyde, K.D.; Bhat, D.J.; Chomnunti, P.; Wen, T.C. Beta-tubulin and Actin gene phylogeny supports *Phaeoacremoniumovale* as a new species from freshwater habitats in China. *MycoKeys* **2018**, *41*, 1–15.
52. Huang, J.E.; Song, H.Y.; Huang, X.G.; Ma, J.; Hu, D.M. *Phaeomonilia aquatica* sp. nov., an aquatic hyphomycete from China. *Mycotaxon* **2018**, *132*, 919–923, doi:10.5248/132.919.
53. Huang, J.E.; Song, H.Y.; Ma, J.; Guan, G.X.; Hu, D.M. *Xylohyphopsis aquatica* sp. nov., a new aquatic hyphomycete from China. *Mycotaxon* **2016**, *131*, 391–394, doi:10.5248/131.391.
